# Real-Time Neuron Detection and Neural Signal Extraction Platform for Miniature Calcium Imaging

**DOI:** 10.3389/fncom.2020.00043

**Published:** 2020-06-26

**Authors:** Yaesop Lee, Jing Xie, Eungjoo Lee, Srijesh Sudarsanan, Da-Ting Lin, Rong Chen, Shuvra S. Bhattacharyya

**Affiliations:** ^1^Department of Electrical and Computer Engineering, University of Maryland at College Park, College Park, MD, United States; ^2^National Institute on Drug Abuse, National Institutes of Health (NIH), Baltimore, MD, United States; ^3^Department of Diagnostic Radiology and Nuclear Medicine, University of Maryland at Baltimore, Baltimore, MD, United States; ^4^Institute for Advanced Computer Studies (UMIACS), University of Maryland at College Park, College Park, MD, United States

**Keywords:** real-time image processing, real-time neuron detection, dataflow, calcium imaging, data stream mining

## Abstract

Real-time neuron detection and neural activity extraction are critical components of real-time neural decoding. In this paper, we propose a novel real-time neuron detection and activity extraction system using a dataflow framework to provide real-time performance and adaptability to new algorithms and hardware platforms. The proposed system was evaluated on simulated calcium imaging data, calcium imaging data with manual annotation, and calcium imaging data of the anterior lateral motor cortex. We found that the proposed system accurately detected neurons and extracted neural activities in real time without any requirement for expensive, cumbersome, or special-purpose computing hardware. We expect that the system will enable cost-effective, real-time calcium imaging-based neural decoding, leading to precise neuromodulation.

## 1. Introduction

Real-time neural decoding predicts behavioral variables based on neural activity data, where the prediction is performed at a pace that keeps up with the speed of the activity that is being monitored. Neuromodulation devices are becoming one of the most powerful tools for the treatment of brain disorders, enhancing neurocognitive performance, and demonstrating causality (Bergmann et al., 2016; Knotkova and Rasche, 2016). A precise neuromodulation system integrates neural activity monitoring, real-time neural decoding, and neuromodulation. In precise neuromodulation, a decoding device predicts a behavioral variable based on neural data streams in real time. Based on the decoding results, neuromodulation parameters such as timing, frequency, duration, and amplitude are changed. Precise neuromodulation systems with closed-loop real-time feedback are superior to the fixed (open-loop) neuromodulation paradigm (deBettencourt et al., 2015; Brocker et al., 2017; Ezzyat et al., 2017).

A recent direct brain stimulation study demonstrated significant advantages of precise neuromodulation over open-loop neuromodulation (Ezzyat et al., 2017). This study applied direct brain stimulation with decoding capability to patients with epilepsy to improve their memory. The study found that stimulation increased memory function only if delivered when the decoding device indicated low encoding efficiency, while stimulation decreased memory function if delivered when the decoding device indicated high encoding efficiency. An open-loop neuromodulation system with a fixed stimulation paradigm may not always facilitate improvement of memory function.

Miniature calcium imaging (e.g., see Ghosh et al., 2011; Kerr and Nimmerjahn, 2012; Scott et al., 2013) is a neuroimaging tool that can observe all cells in the field of view in behaving animals, has high spatial and temporal resolution (single-cell spatial resolution and sub-second temporal resolution), and enables chronic imaging. In this paper, we focus on two-photon calcium imaging. A closed-loop real-time neural decoding system based on miniature calcium imaging will lead to a powerful, precise neuromodulation system. The first step in the development of such a neural decoding system is to have an accurate and fast *Real-time Neuron Detection and Activity Extraction* (*RNDAE*) system. In our context, an RNDAE system takes as input a video stream *S* that is generated by a miniature calcium imaging device, which is mounted on the head of a behaving animal. The output produced by the RNDAE system is a set of neuron masks {*n*_1_, *n*_2_, …, *n*_*m*_} that is detected in *S*, where *m* is the number of detected neurons, along with the neural signal *s*_*i*_(*k*) that is extracted for each neuron *n*_*i*_. The neural signal *s*_*i*_(*k*) gives the neural activity associated with neuron *n*_*i*_ for each input video frame *k*, as represented by the video stream *S*. See Table S2 for the definitions of variables and symbols in this article.

The tremendous rate at which miniature calcium imaging devices produce data imposes major challenges in the design and implementation of an RNDAE system. For example, during 10 min of imaging, such a device generates 1 G of data at a frame rate of 10 Hz. Additionally, intensive processing within and across video frames in the input data stream is required for accurate detection of neurons and extraction of the associated neural signals. Furthermore, since algorithms and hardware platforms relevant to neural signal processing are evolving rapidly, the design of an RNDAE system should be architected in a manner that supports flexible adaptation to different component algorithms and retargeting to different processing devices. These requirements for complex processing on high-rate video data and flexible support for hardware/software design modifications make the development of RNDAE systems a very difficult task.

In this paper, we develop a novel RNDAE system, called the *Neuron Detection and Signal Extraction Platform* (*NDSEP*), which is designed to address the challenges described above. NDSEP provides an experimental platform for neuron detection and neural signal extraction that provides real-time performance and adaptability to new algorithms and hardware platforms. NDSEP also provides a valuable foundation for research and development of precise neuromodulation systems. The architecture of NDSEP is based on principles of signal processing-oriented dataflow models of computation (e.g., see Lee and Parks, 1995; Bhattacharyya et al., 2019).

In dataflow programming, computational tasks can be executed whenever they have sufficient data. This property provides great flexibility to compilers, software synthesis tools, and system designers to coordinate task execution in ways that are strategic with respect to the relevant implementation constraints and objectives. The data-driven semantics of task execution in dataflow is fundamentally different from procedural programming languages, such as C and Java, where the programmer specifies a sequential control flow between tasks in addition to the tasks themselves. This sequential approach to programming hides concurrency between tasks, whereas well-designed dataflow representations expose concurrency explicitly. A trade-off is that dataflow representations can be highly non-intuitive to apply to arbitrary types of applications; however, they have been shown to be well-suited to the broad area of signal and information processing (e.g., see Bhattacharyya et al., 2019).

Motivated in part by its utility for efficient implementation on parallel computing platforms, system design using dataflow methods is widely used for complex signal and information processing applications. The high-level signal flow structure that is exposed by well-designed dataflow models is valuable for design optimization in the context of important metrics, including those related to processing speed, memory management, and energy efficiency (Bhattacharyya et al., 2019). Additionally, dataflow provides a precise, abstract representation of computational modules and the interaction between modules within a given signal processing application. The formal, abstract representation provided by dataflow is of great utility in migrating implementations across platforms and also for efficiently expanding, upgrading, or otherwise modifying an implementation that is targeted to a given platform. Throughout the presentation of NDSEP in this paper, we therefore emphasize the ways in which dataflow techniques are employed to help address the complex and multi-faceted challenges, motivated above, that are involved in RNDAE system development.

The major contribution of our paper is the rigorous application of dataflow-based system design methods to real-time neural decoding. There are many systems, such as CaImAn-CNMF (Giovannucci et al., 2019) and STNeuroNet (Soltanian-Zadeh et al., 2019), for neuron detection, which may achieve higher accuracy than our current implementation. However, these algorithms are not dataflow-based and therefore they do not provide the advantages of expandability, cross-platform portability, and high-level design optimization described above. All of these features are useful for flexible experimentation with and practical deployment of neural decoding methods. The main contribution of this effort can therefore be viewed as the design of an overall system, not just a single component.

## 2. Background and Related Work

In this research, we apply advanced methods for dataflow-based system design to address the challenges identified in section 1 for RNDAE technology. In this section, we first review related work on neuron detection and neural signal extraction, and then we present background on dataflow methods for signal processing system design.

### 2.1. Real-Time Neuron Detection

Neuron detection centers on identifying the source (neurons) in the image field of view (FOV). A straightforward method for neuron detection is to manually delineate neuron masks. This manual labeling process is labor-intensive. For semi-automated/automated neuron detection, a PCA/ICA based method (Mukamel et al., 2009) is proposed. This algorithm first runs PCA to reduce data dimensionality, and then uses ICA to segment data into statistically independent spatial and temporal signals. Constrained nonnegative matrix factorization (CNMF)-based methods for neuron detection are described in Pnevmatikakis et al. (2016) and Zhou et al. (2018). Deep learning-based neuron detection methods are proposed in Apthorpe et al. (2016). Although these semi-automated/automated neuron detection methods are powerful, they are not suitable for real-time applications because of long running time. That is to say, the methods mentioned above are not in real time, which is in contrast with our method, which is in real time and will be described later.

Motion correction is a crucial step for accurate neural detection. For real-time applications, motion correction must be integrated as part of the neural detection and neural signal extraction system, as the input arrives directly without any preprocessing. The motion correction problem can be solved by image registration (Resendez et al., 2016). However, these registration algorithms require a running time on the order of seconds to minutes per frame (Vercauteren et al., 2009). Real-time applications require optimized and efficient motion correction.

### 2.2. Dataflow-Based System Design

Dataflow provides a valuable foundation for the design and implementation of novel signal and information processing systems under complex constraints (e.g., see Bhattacharyya et al., 2019). When dataflow is used as an abstraction for signal processing system design, applications are represented as directed graphs, called *dataflow graphs* (Lee and Parks, 1995). Vertices in dataflow graphs, called *actors*, represent computational tasks, such as digital filters, matrix operations, or image transformations, and each edge represents a first-in, first-out (FIFO) buffer that stores data as it passes from the output of one actor to the input of another. Each unit of data within such a buffer is referred to as a *token*.

Dataflow actors abstract the detailed implementation of the corresponding computational tasks while imposing important constraints on how the actors interface with the surrounding graph, regardless of the implementation. These dataflow interface constraints include two major aspects. First, a dataflow actor can execute (*fire*) only when certain well-defined conditions on the buffers associated with its input and output edges are satisfied. These conditions are typically formulated in terms of the token populations on the buffers—that is, some minimum amount of data is required on each input buffer (to provide the input for the next firing), and some minimum amount of empty space is required on each output buffer (to store the output generated by the firing). When the firing conditions described above are satisfied, the actor (or its next firing) is said to be *enabled*.

Second, when an actor is fired, it must actually produce and consume on each output and input port, respectively, a number of tokens that is consistent with the assumptions that were used to determine that the firing was enabled.

A distinguishing feature of dataflow is that the “program” (dataflow graph) does not specify the order in which actors will execute, nor (in the case of a hardware platform with multiple processors) the processing resource on which each actor is mapped. Instead, the mapping of actors to processors and execution ordering of the actors are left up to the system designer or design tool. The mapping, together with the ordering of actors that share the same processor, is referred to as the *schedule* for the dataflow graph. A general rule of dataflow schedule construction is that an actor can only be fired (executed next in the evolution of a schedule) when it is enabled, as described above.

The schedule typically has a great impact on most or all key implementation metrics, including throughput, latency, and memory requirements. The decoupling of a dataflow graph *G* from the schedule, together with the high-level signal flow structure exposed by *G*, provides great flexibility to designers and design tool developers in constructing schedules. This flexibility is important for optimizing a schedule with respect to the specific constraints, objectives, and processing devices that are relevant to the given application. In this work, we seek to enable and exploit this flexibility by applying dataflow-based concepts consistently throughout the RNDAE system design process.

Formally, a dataflow graph is represented as a directed graph *G* = (*X, E*), where *X* is the set of actors and *E* is the set of edges. For each edge in *e* ∈ *E*, we denote the source and sink vertices of *e* as *src*(*e*) and *snk*(*e*), respectively. Each edge *e* has a nonzero-integer *delay* associated with it, which gives the number of initial tokens that are stored in the corresponding FIFO before the dataflow graph begins execution. A *self-loop* edge is an edge *e*_*s*_ whose source and sink actors are identical (*src*(*e*_*s*_) = *snk*(*e*_*s*_)).

**Figure 1:**
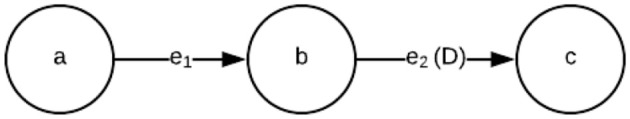
An example of a simple dataflow graph.

Figure 1 shows a simple dataflow graph with three actors (*X* = *a, b, c*), and two edges *e*_1_ = (*a, b*) and *e*_2_ = (*b, c*). The “D” on edge (*b, c*) represents a unit delay. If the delay on an edge exceeds 1, then we typically annotate the edge with “*N* D”, where *N* is the delay of the edge. If the delay is zero, then we omit the “D” symbol, and do not provide any annotation on the edge associated with delay. For example, the absence of a “D” symbol on (*a, b*) in Figure 1 indicates that this edge has no delay.

Self-loop edges are often omitted from drawings of dataflow graphs. However, their presence must be taken into account by some forms of analysis and optimization. For example, self-loop edges in general limit the amount of data parallelism that can be exploited when scheduling a given actor (e.g., see Lin et al., 2018).

For further background on dataflow fundamentals for signal processing systems, we refer the reader to Lee and Parks (1995) and Bhattacharyya et al. (2019). For background on more general foundations of dataflow, we refer the reader to Dennis (1974) and Gilles (1974).

## 3. Proposed Method

Our NDSEP system is developed and tested for use on video streams that are acquired from mice using miniature calcium imaging devices. We especially focus on two-photon calcium imaging. The NDSEP system is therefore suitable for use in monitoring neural activity in real time—for example, to help inform the scientist performing an experiment about how to adapt experimental options so that subsequently acquired data is most relevant to the experiment objectives.

The system design of NDSEP incorporates two distinct modes of operation, which we refer to as the *initialization mode* and *real-time mode*. The purpose of the initialization mode is to optimize system- and actor-level parameters in relation to the image characteristics associated with a given experiment. Calcium imaging data for a given experiment have certain distinctive characteristics that are influenced by the experimental setup, including the imaging devices, neuron types, and specific animal subjects involved. To maximize neuron detection and signal extraction accuracy, it is important to tune, in relation to these distinctive characteristics, certain parameters associated with the neural signal processing algorithms that are employed. Image characteristics that are relevant in this tuning process include the size of the neurons being monitored and the brightness of the firing neurons relative to the background.

For concreteness and for insight into specific optimizations that we applied to facilitate real-time performance, we describe in this section selected details on actor implementations in the current version of NDSEP. These details include, for example, specific OpenCV functions that are applied within the actors and associated parameter settings for these functions. However, we would like to emphasize that the NDSEP framework is independent of any specific approach for implementing algorithms or any specific algorithms for image analysis. For example, one could replace the calls to OpenCV functions with calls to a different library that provides similar capabilities or with customized code that is developed by the actor designer. As another example, one could replace the Neuron Detection actor, which implements the SimpleBlobDetector algorithm, with another actor that implements the Holistically nested Edge Detection (Hed) or MaskRCNN algorithm. The modular, model-based design of NDSEP facilitates use cases such as these for experimentation with alternative algorithms and actor implementations. Such experimentation is useful for gaining insight into trade-offs between neural decoding accuracy and real-time performance, which are critical to the overall utility of a neural decoding system.

In section 4, we evaluate NDSEP using datasets involving both simulated data and real-world data. The real-world dataset is acquired from mouse models. Two-photon calcium imaging was used to image the calcium fluorescence of Anterior Lateral Motor (ALM) cortex. Thus, in the remainder of the paper, we refer to the real data as the ALM dataset. More details about the ALM data we use is given in section 4.

The remainder of this section is organized as follows. First, we provide background on a specific form of dataflow modeling called *parameterized synchronous dataflow* (*PSDF*), which is well-suited to the computational structure of NDSEP. Next, we present the key actors (dataflow-based software components) that are involved in NDSEP. We then present the overall system design for NDSEP, including relevant details of the initialization mode and real-time mode.

### 3.1. PSDF Modeling

A variety of specialized dataflow modeling techniques have been developed for different classes of signal processing applications (e.g., see Bhattacharyya et al., 2019). For design of NDSEP, we apply the PSDF model due to its utility in representing signal processing applications in which dynamic modifications to system parameters play an important role. PSDF enables the joint, dataflow-based modeling of (1) subsystems whose parameters can be modified dynamically (*adapting subsystems*) along with (2) subsystems whose results are used to determine new values of relevant parameters in the adapting subsystems (*controlling subsystems*) (Bhattacharya and Bhattacharyya, 2001).

A number of different variants of dataflow have been developed with an emphasis on supporting dynamic parameter reconfiguration (e.g., see Desnos and Palumbo, 2019). Among these, we apply PSDF because PSDF is well-supported in the software tool, called the lightweight dataflow environment (LIDE) Lin et al., 2017, that we use in this work for dataflow graph implementation. Adapting NDSEP to other forms of dynamic-parameter-integrated dataflow models is an interesting direction for future work in exploring implementation trade-offs.

In the PSDF modeling approach that we use in NDSEP, the system-level dataflow graph is composed of two communicating subgraphs called the *subinit graph* and *body graph*. These graphs are used, respectively, to model the controlling subsystems and adapting subsystems described above. In NDSEP, the body graph represents the core signal processing functionality for neuron detection and activity extraction, while the subinit graph represents functionality for dynamically computing new values for selected parameters in the body graph. In particular, each output port *p* of the subinit graph is associated at design time with one or more ordered pairs ((*A*_1_(*p*), *P*_1_(*p*)), (*A*_2_(*p*), *P*_2_(*p*)), …(*A*_*n*(*p*)_(*p*), *P*_*n*_*p*_(*p*)_(*p*)), where *n*(*p*) is the number of such ordered pairs associated with *p*, each *A*_*i*_(*p*) is an actor in the body graph, and each *P*_*i*_(*p*) is a parameter of actor *A*_*i*_(*p*). When the PSDF graph executes, each iteration of the subinit graph is followed by the transmission of values from each output port *p* to update each parameter *P*_*i*_(*p*) of each actor *A*_*i*_(*p*).

More details on the PSDF-based application model for NDSEP are discussed in section 3.3.

### 3.2. Signal Processing Modules in NDSEP

In this section, we discuss the design of the signal processing actors that are employed in the body graph of NDSEP.

A common approach used in the implementation of the actors in NDSEP is that actors produce and consume pointers to images rather than directly producing and consuming image pixels on their incident dataflow edges. That is, in cases where images are communicated across a dataflow edge *e*, we transfer only a pointer to each communicated image through the FIFO buffer associated with *e* rather than writing and reading the entire image to and from the buffer. The same approach is used when communicating matrices across actors. This approach allows us to adhere to the dataflow principles described in section 2.2 without requiring large overhead for FIFO buffers that carry streams of images or matrices.

The system-level dataflow graph for NDSEP, including all of the actors discussed in this section, is developed using the LIDE tool mentioned in section 3.1. For background on LIDE, we refer the reader to Lin et al. (2017).

#### 3.2.1. Motion Correction

Motion correction is the first step of image processing in NDSEP. In real calcium imaging data taken from moving mice, significant motion can result due to the drift of the implanted imaging device. This kind of shaking in general may result in motion translation as well as slight rotation, thereby distorting the acquired video stream. The goal of motion correction in NDSEP is to remove such motion translation and rotation from image frames.

Through profiling of execution time across different actors in the NDSEP system, we determined early on in the design process that motion correction contributes significantly to overall system execution time. More details on system-level profiling are provided in section 4. Because of the critical role of motion correction in determining overall system efficiency, we applied a significant portion of our design effort to optimizing the accuracy/speed trade-off for this part of NDSEP.

For motion correction, NDSEP utilizes the Enhanced Correlation Coefficient (ECC) algorithm (Evangelidis and Psarakis, 2008) for *motion detection*, which is a core part of motion correction. We selected ECC because it provides parameter settings that give significant flexibility in exploring trade-offs between accuracy and processing speed. Such exploration is useful in the design of RNDAE systems, where the objective is to provide acceptable accuracy in real time rather than maximum accuracy at any cost. ECC is also invariant to photometric distortions in brightness and contrast.

We employ the ECC function provided by the OpenCV library (Demiröz, 2019), and call this function within the LIDE-based actor implementation for the Motion Correction actor.

In addition to using the ECC algorithm, as described above, we apply two major techniques to improve the real-time performance of the Motion Correction actor. First, before comparing frames for motion detection, we downsample the frames by a factor of 1.67 in each dimension so that the number of pixels is reduced to one-quarter of the original pixel count. The downsampled image is currently applied only to the detection process so that any distortion introduced by it is localized to the detection step. Applying downsampling strategically in other parts of NDSEP is a useful direction for future work.

Second, while our motion correction approach takes both translation and rotation into account, we apply rotation selectively, only in cases where translation-based motion correction fails. This optimization is motivated by empirical observations that, in our experimental context, rotation is encountered relatively infrequently in frames that are captured by the neuron imaging device. For example, in the ALM dataset, the rotation frequency detected by NDSEP is 1.62% and the mean and maximum rotation angles are 0.0232 and 0.0733 degrees, respectively. We choose rigid motion correction because of algorithm efficiency for real-time applications. When a single frame is acquired quickly (<50 ms), the influence of motion across the frame is relatively uniform, and a rigid correction can give good results (Thevenaz et al., 1998; Stringer and Pachitariu, 2019). Translation is more common. Furthermore, detection and correction of rotation are more computationally expensive compared to translation. For example, we found that the “Euclidean” mode for the OpenCV ECC function, which detects both translation and rotation, takes on average about three times longer to compute compared to the “Translation Only” mode.

**Figure 2:**
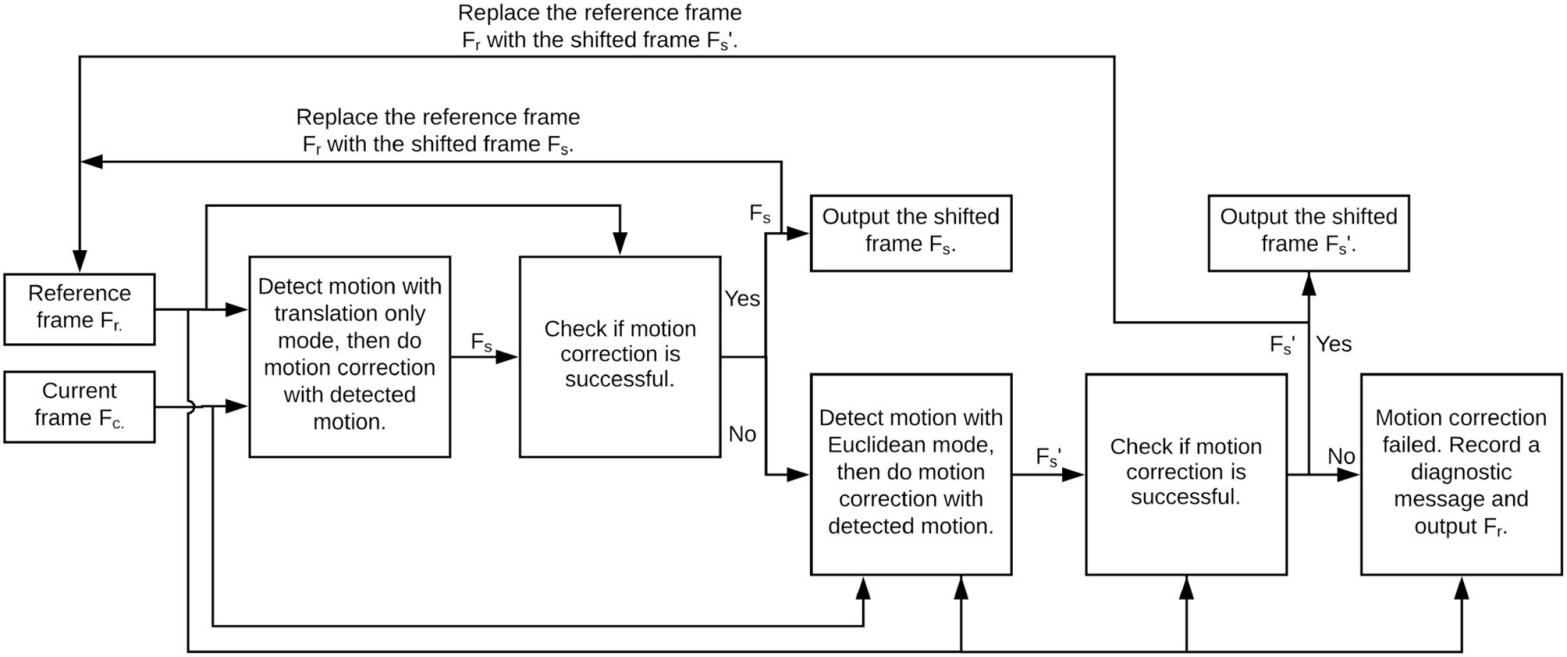
Flowchart for Motion Correction actor operation in NDSEP.

Figure 2 illustrates a flowchart of the optimized motion correction approach in NDSEP, which is based on differences in frequency of occurrence and computational complexity associated with translation and rotation. As illustrated in Figure 2, we first apply motion detection with the Translation Only mode. If motion is detected from this operation, then the current frame *F*_*c*_ is shifted to compensate for the detected translation, and the correlation between the shifted frame *F*_*s*_ and the reference frame *F*_*r*_ is evaluated. On the other hand, if no motion is detected, the correlation is carried out between *F*_*c*_ and *F*_*r*_. If the computed correlation *C*_1_ meets or exceeds a threshold τ_1_, then *F*_*r*_ is replaced with *F*_*s*_ or *F*_*c*_, respectively, *F*_*s*_ is produced on the output edge of the actor, and the current actor firing is complete.

On the other hand, if the correlation *C*_1_ is less than τ_1_, then motion detection for both translation and rotation is applied using the more costly Euclidean mode of OpenCV ECC. If motion is detected from the Euclidean mode, then a shifted version $F_{s}^{\prime }$ of *F*_*r*_ is derived based on the detection result. Then the correlation *C*_2_ between *F*_*r*_ and $F_{s}^{\prime }$ is carried out, where $F_{r}=F_{s}^{\prime }$ if motion was detected from the Euclidean mode, and *F*_*r*_ = *F*_*c*_ otherwise.

Again, a thresholding check, using another threshold τ_2_, is used to determine how to interpret the correlation result. If *C*_2_ ≥ τ_2_ (similar to the case of *C*_1_ exceeding the threshold), then *F*_*r*_ is replaced with $F_{s}^{\prime }$ or *F*_*c*_, respectively; $F_{s}^{\prime }$ is produced on the output edge of the actor, and the actor firing is complete. Otherwise, a diagnostic message is sent to a log file associated with the overall experiment, and *F*_*r*_ is produced on the output edge to complete the firing.

The diagnostic message generated in this last case identifies the input frame index and indicates that motion correction has failed at this index. Such information, which is accumulated in an experiment log file by all relevant actors, can be useful to the system designer for continually improving the robustness of individual actors and the overall system.

The thresholds τ_1_ and τ_2_ defined above, which determine whether to accept the motion correction result or not, are computed adaptively to track any relevant changes in image characteristics. This is due to dynamic variation in the characteristics of calcium imaging frames. For example, some of the datasets are noisy or have contaminated backgrounds. This lowers the average correlation value. For the ALM dataset that we employed in section 4.3, the noise/contamination level is stable over short time periods. During short time periods, the impact of noise/contamination is less significant than the impact of alignment on correlation value. Therefore, in NDSEP, correlation values are only compared with close neighbors. For this purpose, NDSEP stores the 100 most recent correlation values in a queue, which we refer to as the *correlation history queue* (*CoHisQ*). Every time CoHisQ changes, the mean value $\bar{CoHisQ}$ and standard deviation σ_*CoHisQ*_ across all elements in the queue are calculated. Each threshold τ ∈ {τ_1_, τ_2_} is computed from $\bar{\text{CoHisQ}}$ and σ_CoHisQ_ using:

(1) $$\begin{array}{l} \tau =\bar{CoHisQ}-p\left(\tau \right)\times \sigma_{CoHisQ}, \end{array}$$

where *p*(τ) is an empirically defined parameter for each of the two thresholds. In our experiments, we employ *p*(τ_1_) = 2 and *p*(τ_2_) = 10. The threshold values in τ range from approximately [0.3, 0.95] in our experiments resulting in motion correction success.

The threshold computation approach and its associated parameters provide an example of an RNDAE-system design issue for which there are many possible solutions. The modularity and extensibility of NDSEP, based on its dataflow-based foundations, facilitate experimentation across different solutions for such design issues.

#### 3.2.2. Preprocessing

**Figure 3:**
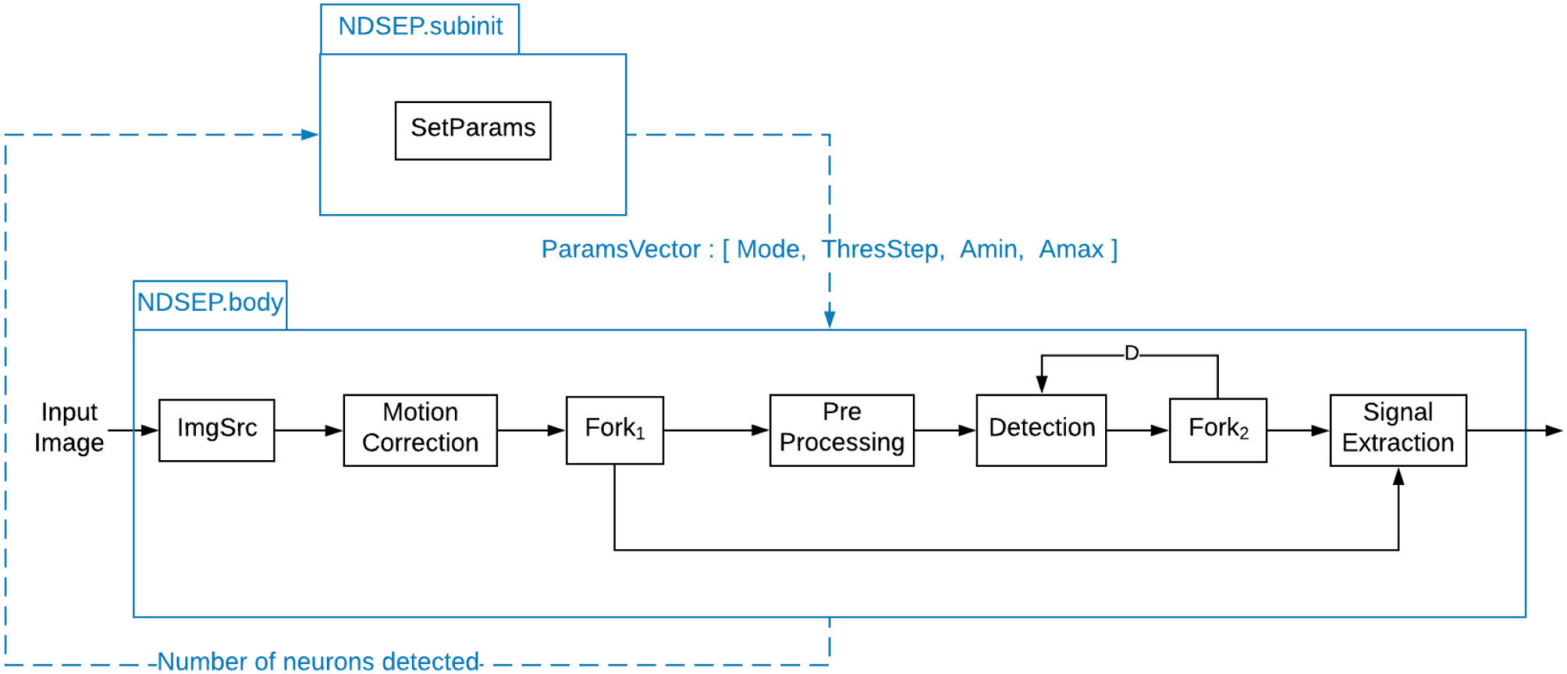
System-level dataflow graph for NDSEP.

The Preprocessing actor is designed to remove image distortion caused by the imaging device and imaging environment. To remove distortion caused by the imaging device, the actor incorporates a Gaussian filter and median filter. Furthermore, background subtraction is used to remove background effects, and image equalization is performed. As described in Figure 3, the output of the preprocessing actor does not affect the neural signal as it only helps to get the positions of neurons. This process helps to eliminate the bright background that results from the firing of neurons in deeper areas of the brain. These deeply located neurons are not of interest in the targeted class of experiments, so it is useful to subtract their potentially strong effect on the image background. To enhance the image's contrast, we normalized the image intensities by using (*I* − *I*_*min*_) × 255/(*I*_*max*_ − *I*_*min*_), where *I* indicates the current pixel's intensity and *I*_*max*_ and *I*_*min*_ represent the maximum intensity and minimum intensity of the image, respectively. As part of the Preprocessing actor, we employed the GaussianBlur and MedianBlur functions from OpenCV. For the GaussianBlur and MedianBlur functions, we employed a filter size of 3 × 3 in order to minimize the possible distortion of the small neurons. The filter size can be reconfigured based on the distortion level and characteristics of the data. Presently, we only consider the possible distortion and removal that might occur to small neuron sizes.

#### 3.2.3. Neuron Detection

The Neuron Detection actor takes an image frame as input, detects the presence of neurons in the image frame, and outputs the position and size of each detected neuron. The output is produced in the form of an *n*_*d*_ × 3 matrix δ, where *n*_*d*_ is the number of detected neurons. Each row in the matrix corresponds to a detected neuron. We refer to δ as a *neuron detection matrix*. For each row index *i*, *n*_*d*_[*i*][1], and *n*_*d*_[*i*][2], respectively, give the *x* coordinate and *y* coordinate for the center of the *i*th detected neuron, and *n*_*d*_[*i*][3] gives the neuron's radius.

For its core computational task, the Neuron Detection actor applies the SimpleBlobDetector function from OpenCV (Demiröz, 2019). The function detects closed contours (“blobs”), which are assumed to outline the detected neurons. Among the contours, the function can filter out the blobs by intensity, size, and shape. The function finds blobs using the parameter thresholdStep, which denotes the minimum intensity difference between the inside and outside of a blob. By using the parameter, it filters out the blobs that have low intensity difference compared to their backgrounds—that is, it removes fewer active blobs. Using the parameters *A*_*min*_ and *A*_*max*_, which are related to the size of blobs to detect, the function computes a set of detected blobs, along with their centers and radii. The parameters *A*_*min*_ and *A*_*max*_ specify the minimum and maximum sizes (in terms of the number of pixels contained) of the blobs to detect. Using parameters for circularity, inertia, and convexity, the function filters out non-neuron-like shapes. The radius of a blob is computed to be the distance between the center of the blob and the furthest point from the center. In this context, a blob can be viewed as a set of connected pixels in an input image that have some minimum intensity (exceed a threshold on the pixel value) and satisfy size constraints that are carefully configured to help ensure that the corresponding image regions represent neurons within the imaged brain region. Since the SimpleBlobDetector function is not for segmentation but for detection, it returns the position of each blob's center and its radius. By using SimpleBlobDetector instead of a segmentation function, we can gain comparable detection results with faster speed.

To this end, the SimpleBlobDetector function is configured to filter blobs by size based on two size-related parameters, which we denote by *A*_*min*_ and *A*_*max*_ (*A*_*min*_ < *A*_*max*_).

The values of *A*_*min*_ and *A*_*max*_ are determined as part of the initialization mode for the NDSEP system. The initialization mode includes an automated training process that configures parameters such as *A*_*min*_ and *A*_*max*_. Another parameter of the SimpleBlobDetector that is configured during the initialization process is the thresholdStep parameter, which controls the step size for determining the set of pixel-intensity thresholds that are used during the blob detection process (Demiröz, 2019). More details on the initialization mode are discussed in section 3.3.

The minThreshold parameter for the simpleBlobDetector function is set to zero in all of our experiments.

After blob detection within a given firing of the Neuron Detection actor, a set of neurons η = μ_1_, μ_2_, …, μ_*k*_ is identified in the input image along with their positions and radii. During real-time operation of the actor, the positions and radii of these neurons are produced as output in the form of a neuron detection matrix, as described above.

During its training process, however, further processing using the set η is performed before producing output. The Neuron Detection actor is equipped with a parameter that is used to select whether it operates in training mode or real-time mode. In NDSEP, Neuron Detection operates in its training mode during a well-defined system initialization phase (discussed further in section 3.3) and then operates for the remainder of the given experiment in its real-time mode.

In the remainder of this section on the Neuron Detection actor, we discuss the further processing that is performed during the training process, after η has been determined.

First, if the current firing is not the first firing within the experiment, the neuron positions in η are compared with those in δ_*p*_, which is the detection matrix derived from the previous actor firing. The previous matrix δ_*p*_ is maintained as a state variable of the Neuron Detection actor. This state variable is maintained and used only in the training mode. By a state variable, we mean a data object that is local to the actor and that persists across firings of the actor. Actor state can be modeled in signal processing dataflow graphs with self-loop edges (e.g., see Zhou et al., 2014).

If the position of a neuron within η is found to be sufficiently close to a neuron position in δ_*p*_, then that neuron is removed from η. In our current design, “sufficiently close” in this context means that the difference in position can be *d* pixels in both the *x* and *y* dimensions. The parameter *d* can be determined by considering how close it should be to be considered as a neuron that has slight motion. That is to say, the user can define a distance criterion such that, if the distance is closer than *d* pixels, the system can regard the neurons as a single neuron, but if two close-together neurons are not closer than *d* pixels, then the neurons will be considered two different, overlapping neurons. After removing all neurons from η that are sufficiently close to corresponding neurons in δ_*p*_, the remaining neurons in η are interpreted to be *newly discovered* neurons in the training process. Thus, all of the remaining neurons in η are appended to those in δ_*p*_. The resulting δ_*p*_ may be unchanged from the previous firing (if there were no newly discovered neurons), or it may contain one or more new neurons. The resulting δ_*p*_ is produced as the output of the training mode firing, and it is also retained as the updated value of the corresponding state variable in the actor.

#### 3.2.4. Signal Extraction

Each firing of the Signal Extraction actor takes as input a motion-corrected image frame *F*_*mc*_ and a neuron detection matrix δ that gives the positions and radii of the neurons that have been detected in *F*_*mc*_. The output of the firing is a vector β that gives the relative intensity of each detected neuron.

Each *i*th element of β corresponds to a distinct neuron and is calculated as β[*i*] = (*F*(*i*)(*F*_*mc*_) − *F*0)/*F*0), where *F*(*i*)(*F*_*mc*_) is the average intensity (average pixel value) across all pixels in the circle centered at (δ[*i*][1], δ[*i*][2]) and having radius δ[*i*][3] in the *F*_*mc*_th image frame. To calculate F0, we followed (Romano et al., 2017), using the average ROI intensities across a window of time that immediately precedes a particular experimental event.

Throughout a given experiment, the Signal Extraction actor produces a sequence of vectors β_1_, β_2_, …, β_*L*_, where *L* is the total number of image frames in the input video sequence for the experiment (excluding the frames used for system initialization/training). Each β_*i*_ is a ν-element vector, where ν is the total number of neurons that have been detected throughout the training process for the Neuron Detection actor. The sequence β_1_[*i*], β_2_[*i*], …, β_*r*_[*i*] thus provides a sampled representation of the relative pixel intensity for each *i*th detected neuron (1 ≤ *i* ≤ ν).

### 3.3. System Design

Figure 3 shows how the different actors described in section 3.2 are integrated into the dataflow graph for the NDSEP system. The dataflow graph is based on the PSDF model of computation (see section 3.1). As discussed earlier in this section, the system has two distinct modes of operation—the initialization mode (also known as the training mode) and the real-time mode.

The initialization mode is used to configure selected actor parameters in the body graph using a set of *training frames*. The training frames are captured from a calcium imaging device that is implanted within a given animal subject. The resulting set of optimized parameters is then applied to perform accurate real-time processing during neuron image acquisition and analysis experiments involving the same device and animal subject. This real-time processing corresponds to the real-time mode of NDSEP. The set of frames that is processed when in the real-time mode for a given experiment is referred to as the set of *analysis frames*. For more details, see sections 3.2.3, 3.3.2.

#### 3.3.1. Auxiliary Actors

All of the core signal processing actors in Figure 3 have been discussed in section 3.2. Four additional actors—namely, the actors labeled ImgSrc, Fork_1_, Fork_2_, and SetParams—are also used, as shown in Figure 3.

Each firing of the ImgSrc actor reads the next image from the input video sequence from disk into memory, and outputs a pointer to the memory block that contains the image. This disk-based interface is used in our current NDSEP prototype, since our focus is on functional validation and on optimizing trade-offs between accuracy and real-time performance. For integration into a complete experimental system, the ImgSrc actor can readily be replaced by an actor that provides direct software interfacing with the image acquisition device.

The actors labeled Fork_1_ and Fork_2_ are *fork* actors, also referred to as *broadcast* actors. Each firing of a fork actor consumes one token on its input and produces a copy of the token on each of its outputs. Since images and matrices are communicated by reference (through pointers) in NDSEP (see section 3.2), the fork actors require minimal execution time compared to the core signal processing modules in the system.

The fourth auxiliary actor, SetParams, is discussed in section 3.3.2.

#### 3.3.2. Adapting the Neuron Detection Actor

The SetParams actor is used during the initialization process to adaptively optimize parameters of the Neuron Detection actor. The objective is to calibrate the selected parameters to the given calcium imaging device and animal subject so that neuron detection accuracy is enhanced compared to that with the use of generic parameter settings. The parameters are adapted progressively as the training frames are processed in the initialization mode. Specific parameters that are configured by the SetParams actor are the *A*_*min*_, *A*_*max*_, and thresholdStep parameters for neuron detection (see section 3.2.3). Before running the initialization mode, we manually “pre-initialize” these parameters by considering the size of the input image and rough size of the neurons. The initialization mode then uses the pre-initialized parameter values as a starting point and optimizes the three values through an iterative process (see section 3.2.3).

Many different approaches for adapting neuron detection processes can be envisioned for use in NDSEP. Presently, we use a relatively simple adaptation approach that progressively loosens the filtering constraints of the blob detector used in the Neuron Detection actor. The constraints are loosened until a pre-determined target number *T*_*n*_ of neurons is detected. Presently, we use the empirically determined value *T*_*n*_ = 5. Incorporating more sophisticated parameter adaptation processes into NDSEP is a useful direction for future work.

We conducted some simple experiments to help validate our current adaptation approach. With the simulated data, when we tried an approach that progressively tightens the constraints, we observed more false positives than our proposed approach, which progressively loosens the constraints. For example, with the most noisy set of simulated data, which is described in 4.1, we observed 11% more false positives with progressive tightening compared to our proposed approach. In this dataset, the progressive tightening approach also led to a few false negatives, whereas there were no false negatives resulting from our proposed approach.

#### 3.3.3. Real-Time Mode

For the real-time mode of NDSEP, the iteration count for the body graph is set to the total number of analysis frames. Thus, the subinit graph is effectively disabled through the duration of running in real-time mode. This is because the body graph continues executing for the specified number of iterations before control returns to the subinit graph, at which point the neural decoding process terminates.

In real-time mode, each analysis frame is processed by correcting motion, detecting neurons, and then extracting relative pixel intensities for each neuron. The relative pixel intensities are used, as described in section 3.2.4, to populate the vector elements for the next time step in the extracted signals for the neurons.

The output of the real-time mode is the sequence of vectors β_1_, β_2_, …, β_*L*_, where *L* is the number of analysis frames. This sequence encapsulates a sampled version of the signal extracted for each neuron. The sequence can be saved for subsequent off-line analysis or connected to another computational subsystem for further real-time processing, as would be the case if NDSEP were embedded within a precise neuromodulation system.

## 4. Experiments

In this section, we present results obtained through experiments using the proposed platform, NDSEP. We first present experiments involving simulated data and then experiments involving real data. The experiments involving real data include results on neural imaging data that has already been processed with motion correction and also results on “raw imaging data” (without motion correction already applied).

### 4.1. Simulated Data

In this experiment, simulated calcium imaging datasets were used to assess the proposed platform because the simulated data had ground-truth. The simulation described interactions among a set of leaky integrate-and-fire neurons with additive noise. The neuron model (Gütig and Sompolinsky, 2006) is as follows:

(2) $$\begin{array}{l} \frac{dV}{dt}=\frac{V_{rest}-V}{\lambda }+\theta \times \lambda \times \left(-0.5\right)\times \epsilon , \end{array}$$

where *V* is the membrane potential, *V*_*rest*_ is the rest potential, ϵ is a Gaussian random variable with mean 0 and standard deviation 1, λ is the membrane time constant, and θ is a parameter to control the noise term. Spikes received through the synapses cause changes in *V*. A neuron fires if *V* is greater than a threshold. After firing, a neuron cannot generate a second spike for a brief time (refractoriness). Such a neuron model can represent many kinds of postsynaptic potentials or currents described in the literature (Brette et al., 2007).

Our simulation included 100 neurons. We divided these 100 neurons into two groups: group A and B. Neurons in group A have no parent nodes, while neurons in group B have one or two neurons in group A as parent nodes. If a parent node fires, the membrane potential of the target node will increase by *w* = 0.2. This simulation represented a scenario in which neurons in group A were responsive to external stimulus, and firing of neurons in group A facilitated firing of neurons in group B. We generated simulated spike trains with 1,800 time points.

One hundred neuron masks from the Neurofinder 00 dataset (Peron et al., 2016) were chosen as ground-truth neuron masks. For a given frame *t*, if neuron *i* fired, then the intensities of pixels inside neuron mask *i* were set to be 128. After this, we performed exponential smoothing to simulate calcium signal decay. jGCaMP7f, which is a calcium sensor, has a decay half-life of around 265 ms (Dana et al., 2019). To simulate imaging jGCaMP7F using a two-photon microscope at 30 Hz, the decay half-life in our simulation was set to 8 frames. To simulate motion, we introduced a global shifting in the *x* and *y* axes. The random translation motion in *x* or *y* followed a uniform distribution in [−10, 10]. Also, to simulate rotation in frames, datasets were generated with different random rotation ranges and occurrence probabilities. We used *P*_*rot*_ to denote the rotation occurrence probability, and α_*rot*_ to denote the rotation range. For example, if α_*rot*_ = 5.16 and *P*_*rot*_ = 10, then rotation within [−5.16^*o*^, 5.16^*o*^] is randomly applied to the simulated data, with a rotation occurrence probability of 10%.

**Figure 4:**
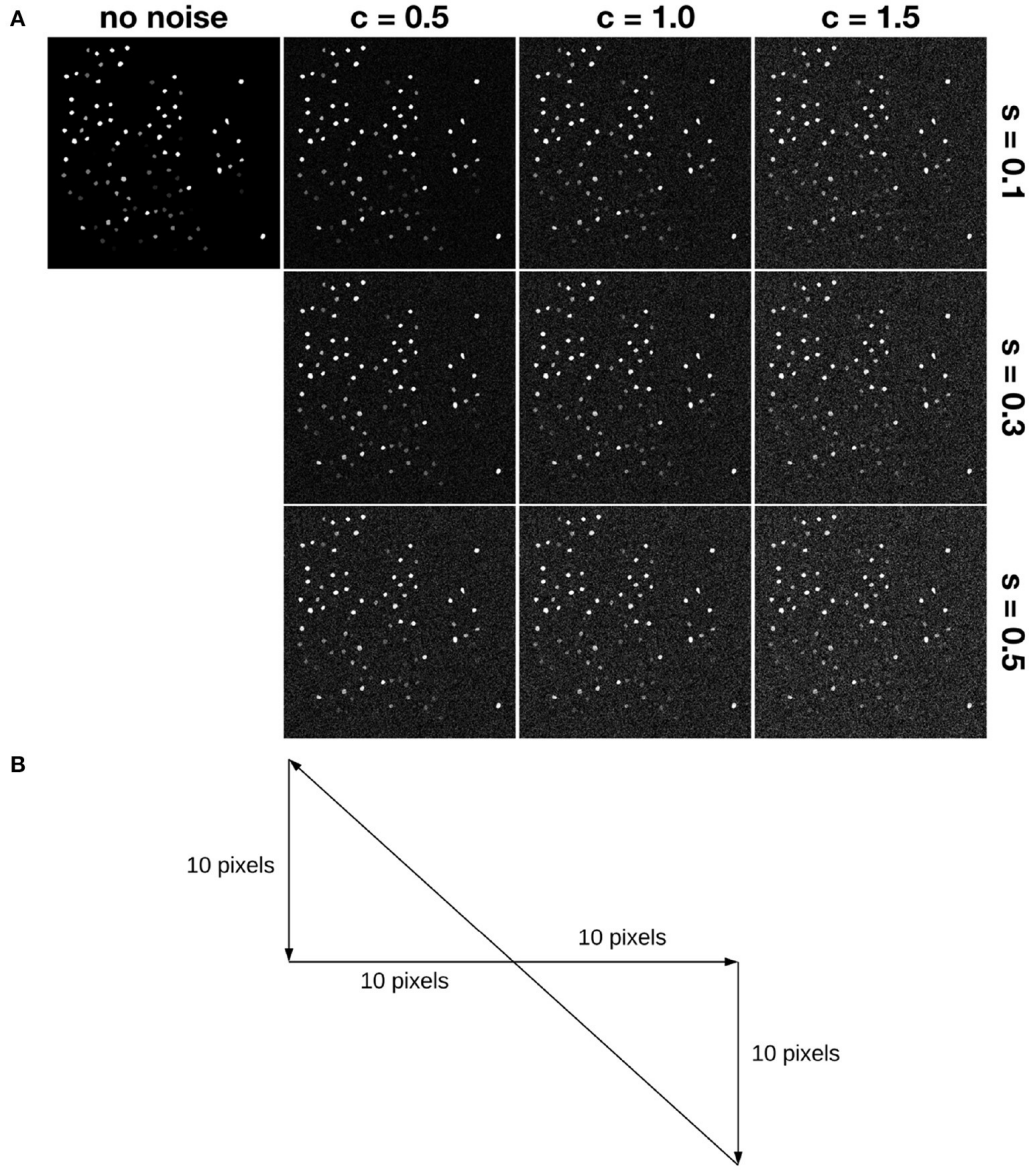
Simulation of motion and noise. **(A)** Shows simulated noise. Here, “s” denotes the relative shot noise level, while “c” denotes the relative colored (red) noise level. For example, s05c15 denotes the noise case in the lower right corner. **(B)** Shows a trajectory simulating slow and constant drift. Each frame moves 1 pixel along the path. For example, assume the first frame has x,y-position (0,0), then the second one is at (1,0), the third one is at (2,0), etc.

In addition to the random translation motion, three kinds of drift are simulated. A slow and constant drift is simulated according to the trajectory shown in Figure 4B. Motions are simulated around the ground truth position within 10 pixels, following the same range that we used for random translation motion. Slow and constant drift was incorporated by moving the frame 1 pixel at each time step in the same direction until it hits the boundary, which is taken to be 10-pixels in the *x* or *y* direction away from the ground truth. Then the direction of the drift changes according to the trajectory. We simulate small and large drift by controlling the range of random translation motion. Motion in *x* or *y* follows a uniform distribution in [−3, 3] or in ([−10, −7]⋃[7, 10]) to simulate small drift and large drift, respectively.

Then we added temporally autocorrelated zero mean noise with standard deviation σ = 0.4 (Svetunkov, 2019) as well as shot noise with zero mean and σ = 1.0 (Pilowsky, 2019) to some of the simulated datasets, called noisy simulated datasets (NoiseSim). All noisy simulated datasets had *P*_*rot*_ = 25 and α_*rot*_ = 6.3153, along with the same global translation motion described above, as shown in Figure 4.

We applied the proposed system to the simulated data. The system was evaluated in terms of neuron mask detection accuracy, signal-to-noise ratio, and running time. For neuron mask detection accuracy, because ground-truth neuron masks were available, we compared each detected neuron mask with the corresponding ground-truth neuron mask, and calculated the recall (the fraction of matched pixels divided by the number of pixels in the ground truth) and precision (the fraction of matched pixels divided by the number of pixels in the detected neuron mask).

For signal-to-noise ratio, for each detected neuron ϕ, we first correlated the detected Δ*F*/*F* of ϕ with the ground-truth spike trains. Given an image frame *I* and some region *r* (a connected subset of pixels) in the frame, Δ*F*/*F* is a measure of the relative pixel intensity in *r* relative to a baseline. The metric is similar to that used for elements of vector β defined in section 3.2.4. Here, *F* represents the baseline pixel intensity, and Δ*F* = *R*−*F*, where *R* is the average pixel intensity for all pixels in *r*. When Δ*F*/*F* is used in the context of a neuron, the region *r* consists of all pixels contained in the neuron. We then calculated the average correlation coefficient *R*_*s*_ between the neuron time course and the ground-truth spike trains across all neurons. Next, for each neuron ϕ, we randomly selected a region of the image frame with a size close to the size of ϕ. For each randomly selected region *r*, we correlated Δ*F*/*F* of *r* with the ground-truth spike train of ϕ. This yielded a correlation coefficient ρ(ϕ). Then we calculated the average correlation coefficient across all ϕ—that is, the average value of ρ(ϕ). To avoid selection bias in choosing random regions, we repeated the above process 1,000 times and calculated the average value *R*_*n*_. The signal-to-noise ratio was then computed as 10log_10_(*R*_*s*_/*R*_*n*_).

The motion-corrected images were compared with the ground truth images. The ideal case here is that the Motion Correction actor detects the ground-truth *x* and *y* motion along with the rotation angle. Three matrices are used to evaluate the performance of motion correction. For each dataset, *M*_*x*_, *M*_*y*_, and *M*_*rot*_ denote *x*-displacement, *y*-displacement, and angle error, respectively. *Rate*_*fail*_ denotes the failure rate of motion correction. When motion correction fails, the frame is not motion-corrected (see Section 3.2.1).

Table 1 shows our measured results for motion correction with α_*rot*_ = 3.43 and different values of *P*_*rot*_. Table 2 shows results with *P*_*rot*_ = 40 and different values of α_*rot*_. In Table 1, we see that as *P*_*rot*_ increases, the error also increases. However, the mean errors of *x*-displacement and *y*-displacement are very small, about 1 pixel in each dimension. The mean rotation error *mean*(α_*rot*_) is close to zero. Although some rotations are not detected (the rotation detection rate is not 100%), such cases are rare. As Table 2 shows, only when α_*rot*_ reaches 7.46^*o*^ does the motion correction failure rate begin to rise in some sparsely occurring cases (1.56% failure rate). However, from our observations, such a large value of the rotation angle is rare in practice. Table 3 shows the performance of NDSEP in noisy situations. As the noise level increases, the *x*, *y*, and angle detection error increases. Even in the s05c15 case, which includes frames that contain large amounts of noise, motion correction still performs effectively. The mean error of *x, y*-displacement remains consistently around 1 pixel, while the mean rotation error is also comparable to the no-noise case. Compared with the average neuron size in simulated data, which has a 6.8387 pixel width and a 6.7634 pixel height, the 1 pixel *x, y*-displacement means NDSEP motion correction is effective for simulated data.

**Table 1:** Motion correction accuracy with different *P*_*rot*_ values without noise.

***P*_*rot*_(*%*)**	**0**	**5**	**10**	**15**	**20**	**25**	**30**	**40**	**50**
*mean*(*M*_*x*_)	0.8002	1.1696	0.9322	0.9911	0.6672	1.1643	1.0612	1.0956	1.0225
*max*(*M*_*x*_)	1.6443	1.9522	1.8845	1.7710	1.9838	1.7457	1.9203	1.9470	1.9464
*mean*(*M*_*y*_)	0.8145	0.7509	0.7655	0.7720	0.7710	0.7903	0.7476	0.7395	0.7146
*max*(*M*_*y*_)	1.1627	1.2483	1.3923	2.6277	1.4583	1.4413	1.3937	1.4621	1.4736
*mean*(*M*_*rot*_)(× 10^−4^)	0.0657	0.2590	0.2666	0.3568	0.5596	0.5988	0.9873	1.2820	2.0379
*max*(*M*_*rot*_)	0.0041	0.0056	0.0042	0.0095	0.0091	0.0067	0.0068	0.0038	0.0040
*R*_*fail*_(%)	0.5	0.44	0.33	0.38	0.33	0.38	0.22	0.33	0.06

**Table 2:** Motion correction accuracy with different α_*rot*_ values without noise.

***sin*(α)**	**0.06**	**0.09**	**0.11**	**0.13**	**0.15**	**0.17**
α	3.4398	5.1636	6.3153	7.4696	8.6269	9.7861
*mean*(*M*_*x*_)	1.0956	1.0257	0.9840	0.9125	1.1493	0.5187
*max*(*M*_*x*_)	1.9470	1.8579	1.8476	2.7967	2.4186	1.1522
*mean*(*M*_*y*_)	0.7395	1.8579	0.7586	0.7543	0.7357	0.8116
*max*(*M*_*y*_)	0.7395	1.8579	1.3687	1.3776	1.3771	1.1597
*mean*(*M*_*rot*_)(× 10^−4^)	1.2820	1.4704	0.8297	0.6719	0.7182	0.6089
*max*(*M*_*rot*_)	0.0038	0.0045	0.0035	0.0030	0.0041	0.0039
*R*_*fail*_(%)	0.33	0.27	0.33	1.56	3.80	7.16

**Table 3:** Motion correction accuracy on simulated noisy datasets (*P*_*rot*_ = 25, α_*rot*_ = 6.3153) with different noise levels.

**Noisy case**	**s01c05**	**s01c10**	**s01c15**	**s03c05**	**s03c10**	**s03c15**	**s05c05**	**s05c10**	**s05c15**
*mean*(*M*_*x*_)	0.7605	1.0310	1.2810	0.5778	1.4274	1.1609	1.1774	1.1628	1.2028
*max*(*M*_*x*_)	1.7837	2.2656	2.8494	2.3209	2.9944	2.5531	2.7935	2.3158	2.1030
*mean*(*M*_*y*_)	0.9026	0.8362	0.9485	0.8211	0.9484	0.9668	0.8385	0.9608	0.8041
*max*(*M*_*y*_)	1.6227	1.8998	2.2747	1.3876	1.6665	2.1496	2.2238	2.1455	1.6457
*mean*(*M*_*rot*_)(× 10^−4^)	0.6755	0.9516	1.1771	0.7090	1.1146	2.3755	1.5048	1.2410	1.8007
*max*(*M*_*rot*_)	0.0049	0.0042	0.0050	0.0060	0.0081	0.0082	0.0071	0.0080	0.0079
*R*_*fail*_(%)	0.56	0.56	0.83	0.89	1.50	0.50	0.67	0.89	0.33

To further evaluate the motion correction process in NDSEP, slow and constant drift is added to the no-noise case s00c00 and to noisy cases s01c05 and s05c15. *R*_*fail*_(%) is 5, 2.11, and 4.33, respectively, for these three cases; *mean*(*M*_*x*_) is 0.6518, 0.8707, and 0.7366, respectively, and *mean*(*M*_*y*_) is 1.4400, 1.4121, and 1.1849, respectively. The results shown above are comparable with the random motion drift cases in Tables 1, 2, indicating that NDSEP-Motion Correction is able to correct slow and constant drift. Similarly, small and large drift are applied to the no-noise case s00c00 and noisy cases s03c10 and s05c15. For small drift in cases s00c00, s03c10, and s05c15: *R*_*fail*_(%) is 0 for all three cases, *mean*(*M*_*x*_) is 2.28, 0.85, and 0.82, and *mean*(*M*_*y*_) is 0.41, 0.84, and 0.70, respectively. For large drift: *R*_*fail*_(%) is 4.5, 0.83, and 26.2, *mean*(*M*_*x*_) is 0.61, 0.89, and 1.17, and *mean*(*M*_*y*_) is 1.45, 1.73, and 1.06, respectively. For large motions with intensive noise, the failure rate of 26.2% is higher than in other cases, while in terms of successful correction output, *mean*(*M*_*x*_) and *mean*(*M*_*y*_) remain comparable to the small drift cases. We anticipate that image frames with such intensive noise and motion do not often occur in practice. In the clear small motion case, 2.28 is a little bit higher than the others, but it is still smaller than the half size of the neurons, which is about 7 pixels, in the simulated dataset.

From the results described above, we conclude that the NDSEP Motion Correction can accurately detect motion translation and rotation—with or without the presence of noise—in most cases.

**Figure 5:**
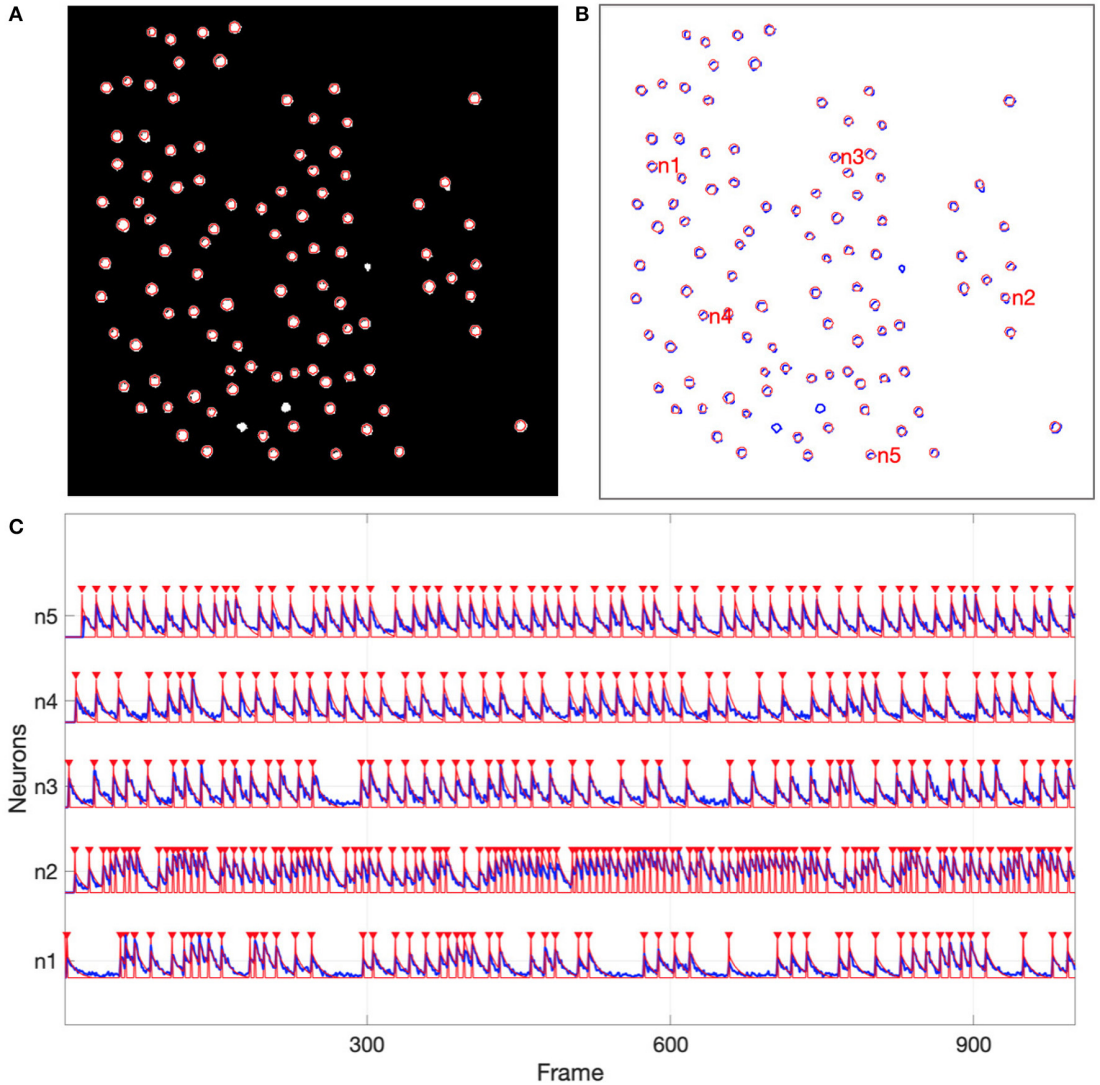
Neuron detection results from the simulated data. **(A)** Shows detected neurons (red-perimeter circles) overlaid on the mean map of all frames in the simulation-derived dataset; **(B)** Shows ground truth neurons with blue perimeters and detected neurons with red perimeters; **(C)** Shows representative neural signals (blue) extracted from the simulated data and ground truth spikes (red).

For the simulated data, all 97 of the active neurons were detected by NDSEP. Three neurons should not be detected, since all three of these were inactive for the duration of the image sequence. In Figures 5A,B, the ground truth neurons are depicted as bright blobs, which are overlaid on the mean map of all 1,800 frames in the simulation-derived dataset in the case of s03c05. Since different neurons have different firing rates, they generally appear with different levels of brightness in the mean map. Each circle with a red perimeter in Figure 5 represents a detected neuron.

Figure 5C shows signals that have been extracted by NDSEP for five randomly chosen neurons. The figure also shows the corresponding ground truth signals. The signals shown in red correspond to spike events. These signals have a value of 1 when the corresponding neuron fires and 0 when the neuron is not firing. Blue signals indicate Δ*F*/*F* values for the detected neurons. From the results in Figure 5, we see that NDSEP accurately detected the spike events. For these results, we computed *R*_*s*_ = 0.354, *R*_*n*_ = 0.000011, and 10log_10_(*R*_*s*_/*R*_*n*_) = 45.08.

### 4.2. Neurofinder Data

We also performed experiments using the Neurofinder collection of datasets (Peron et al., 2016). These datasets represent publicly available, real calcium imaging data that has already been preprocessed with motion correction. The Neurofinder data provide ground truth for the position of each neuron. There are five datasets in the Neurofinder database. The first one (Dataset 00) contains segmented neurons using fluorescently labeled anatomical markers. It is possible that neurons are not firing in Dataset 00 but are still labeled. This is inappropriate for neuron detection based on neural activity. Datasets 02 and 04 have around 8–41% potentially mislabeled neurons (Soltanian-Zadeh et al., 2019). Among the five datasets, 00, 02, and 04 are not suitable for evaluating the neuron detection performance of NDSEP. Therefore, we used Datasets 01 and 03 for our experiments. See Table S1 for details about the Neurofinder data.

**Figure 6:**
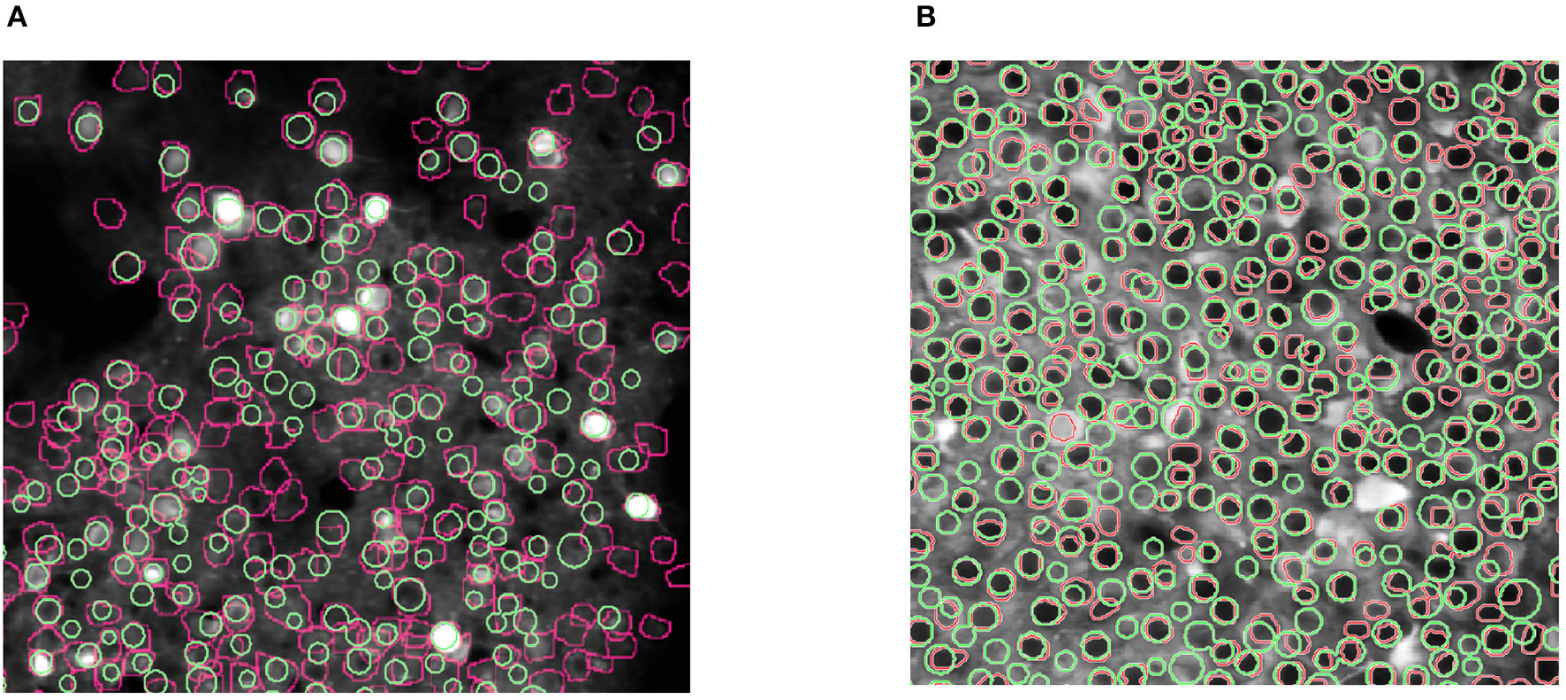
Results of experiments with the two Neurofinder datasets: **(A)** shows results from Dataset 01, and **(B)** shows results from Dataset 03. The ground truth regions are bounded with red perimeters, and the results from NDSEP are bounded with green perimeters.

**Figure 7:**
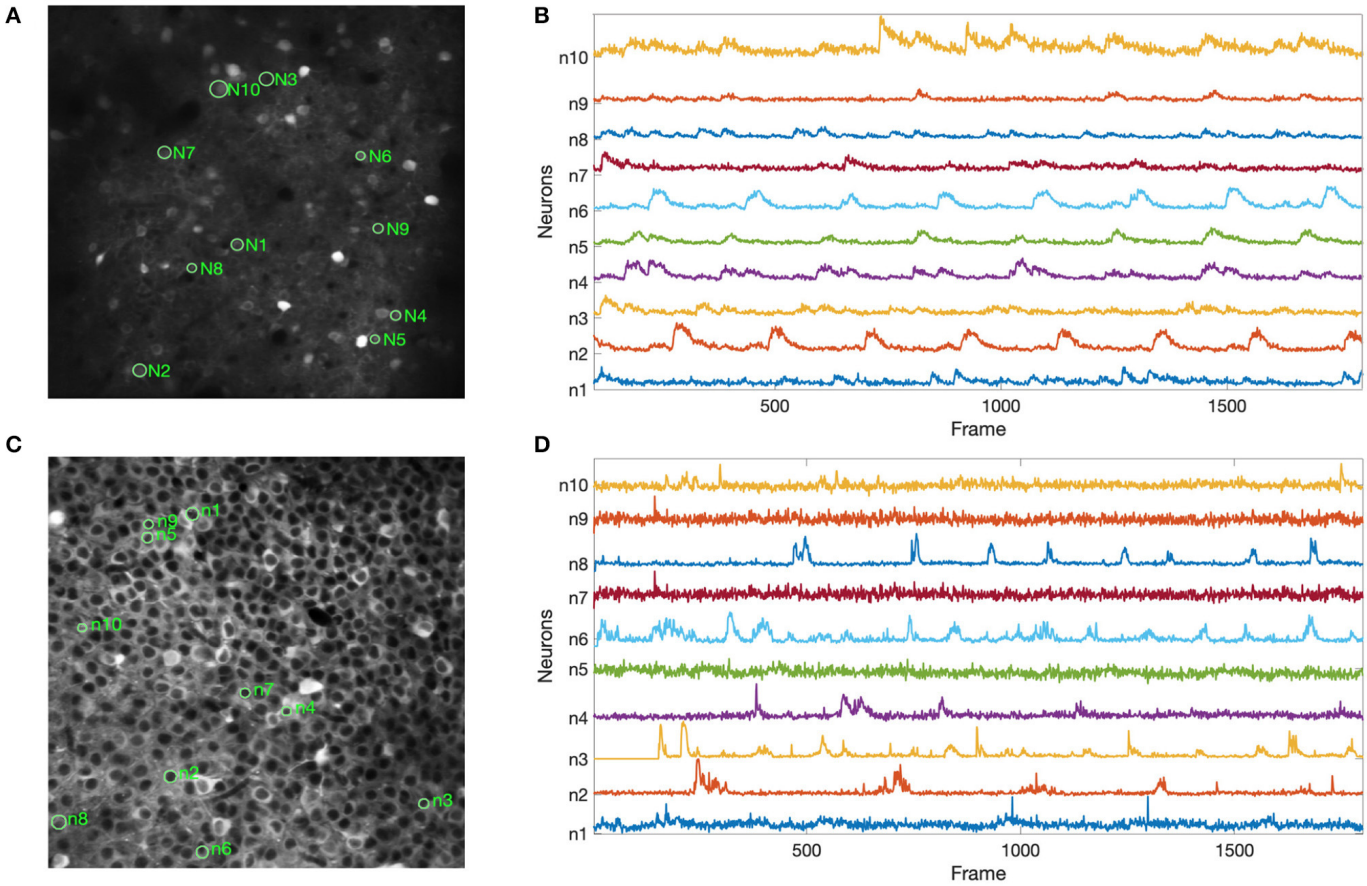
Results using the Neurofinder data for extracted signals from randomly selected neurons: **(A,C)** show the locations of the randomly selected neurons from Dataset 01 and Dataset 03, respectively; **(B,D)** show the signals that were extracted by NDSEP from each neuron. Ten neurons were randomly selected from each of the two datasets.

Figure 6 shows the results of our experiments with the two Neurofinder datasets. The results show that NDSEP is effective at detecting relatively active neurons. The extracted signals for ten randomly selected neurons from each dataset are plotted in Figure 7. Most of the signals in Figure 7B exhibit the signal characteristics that are described in Resendez et al. (2016). Technically, these experiments pertain to the combination of the Preprocessing and Neuron Detection (PND) actors of NDSEP since the experiments do not involve motion correction (the Neurofinder input data are already motion-corrected) or signal extraction. We refer to this actor combination concisely as NDSEP-PND.

Next, we report the precision, recall, and F1 scores achieved by NDSEP-PND across each of the two Neurofinder datasets. The precision is the fraction of true neurons (true positives) detected among detected neurons. The recall is the fraction of actual neurons that are detected. The F1 score is defined as the harmonic mean of precision and recall: F1sore = 2 × (*u* × *v*)/(*u* + *v*), where *u* is the precision and *v* is the recall. For Dataset 01, the precision, recall, and F1 scores are 0.6431, 0.5275, and 0.5848, respectively. For Dataset 03, the precision, recall, and F1 scores are 0.7166, 0.7259, and 0.7212, respectively.

The results on the Neurofinder datasets demonstrate that NDSEP-PND has an accuracy that is comparable with the top five neuron detection algorithms from the comparative experimental study reported on in Klibisz et al. (2017). These top five previously developed algorithms are HNCcorr Spaen et al. (2017), Sourcery, UNet2DS, Suite2p (Pachitariu et al., 2017) + Donuts (Pachitariu et al., 2013), and HNCcorr (Spaen et al., 2017) + Conv2din. Of the six algorithms (the five previously developed ones together with NDSEP-PND), the result of NDSEP-PND has the fifth-highest accuracy (in terms of recall and precision) for Dataset 01, and for Dataset 03, NDSEP-PND also has the fifth-highest accuracy. At the same time, NDSEP-PND achieves real-time performance, while the other five methods are not real-time systems. This is a critical advantage of NDSEP-PND in the context of our work. Also, in relation to the other state-of-the-art algorithms in the Peron et al. (2016) challenges, Kirschbaum et al. (2019) (recall = 0.56, precision = 0.85, F1 = 0.67 for dataset 01), and Soltanian-Zadeh et al. (2019) (recall = 0.65, precision = 0.57, and F1 = 0.61 for dataset 01, and recall = 0.56, precision = 0.54, F1 = 0.55 for dataset 03), NDSEP-PND outputs comparable results for both datasets.

### 4.3. Anterior Lateral Motor Cortex Data

As a representative real-world application, an Anterior Lateral Motor Cortex (ALM) dataset Li et al. (2015) is used to evaluate NDSEP. This dataset includes 11,189 frames of calcium imaging that record the anterior motor cortex in mice while the mice are performing a tactile delay-response task. The motion correction failure rate *Rate*_*fail*_ of NDSEP on the ALM dataset is 0.

**Figure 8:**
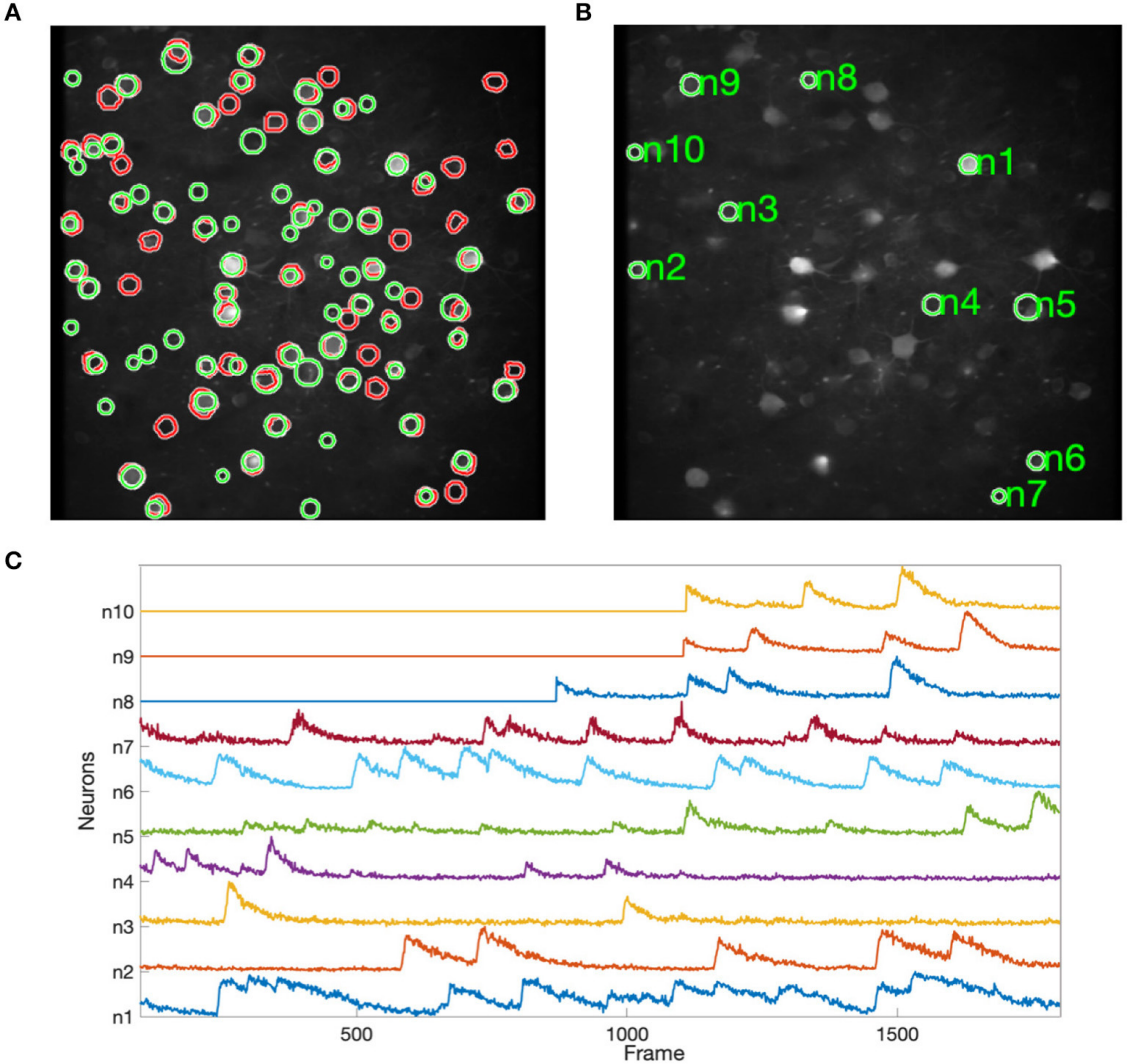
Results for ALM data: **(A)** shows neuron masks in regions with green perimeters, with ground truth in red, overlaid on the mean map of all frames; **(B)** shows randomly selected neurons among the correctly detected result; **(C)** shows the signals that were extracted from selected neurons in **(B)**.

Figure 8 shows the results of applying NDSEP to the ALM dataset. Figure 8A shows the detected neuron masks overlaid on the mean signal map. We randomly picked 10 neurons (Figure 8B) and plotted Δ*F*/*F* (Figure 8C). Δ*F*/*F* captures the characteristics of the neural activity. A neuron is detected only after it fires at least once. For example, Δ*F*/*F* values of neurons 8, 9, and 10 were 0 until their first spiking activity began. In this experiment, NDSEP neuron detection detects 50 neurons. Compared with the ALM ground truth mask, which has 69 neurons, the recall and precision of NDSEP-based neuron detection are 72.46 and 69.44%, respectively. Also, the F1 score is 70.92%.

### 4.4. Execution Time Measurements

Table 4 shows measured execution times for all of the core signal processing actors in NDSEP as well as the ImgSrc actor. The total running time is measured by using time calculation functions. The times shown in Table 4 are the single frame execution times, which are calculated by: Single_Frame_Time = Total_Processing_Time/#Frames_in_Dataset. The mean and standard deviation of the execution time is calculated by repeating the associated experiment 10 times under the same conditions. All execution time measurements are shown in milliseconds (ms). Only the SetParams and fork actors are not considered in these execution time experiments. SetParams is used only in the initialization mode of the application and not in real-time mode. Thus, the execution time of the SetParams actor is not relevant to real-time performance. The fork actor is excluded because it is of minimal complexity and has a negligible impact on overall performance.

**Table 4:** Measured execution times for different actors in NDSEP.

**Data**	**ImgSrc** **(ms)**	**Motion correction (ms)**	**Pre-processing** **(ms)**	**Detection** **(ms)**	**Signal extraction (ms)**	**Total execution time (ms)**
Simulated (400 × 400) 97 neurons .png by NDSEP (Image acquisition rate: 10 Hz)	1.18 (std:0.04)	12.98 (std: 0.84)	0.66 (std: 0.02)	2.72 (std: 0.09)	4.49 (std: 0.14)	22.04 (std:0.87) (45.37 Hz)
Simulated (400 × 400) 97-neuron .png by SIFT	–	131.11 (std: 0.9729)	–	–	–	–
Simulated (400 × 400) 97-neuron .png by NoRMCorre-Rigid	–	21.66 (std: 0.31)	–	–	–	–
Simulated (400 × 400) 97-neuron .png by NoRMCorre-Nonrigid	–	261.12 (std: 12.75)	–	–	–	–
Simulated (400 × 400) 97-neuron .png by CNMF	–	–	–	27.9382 (std: 0.1476)	–	–
Neurofinder 01 (512 × 512) 345-neuron .tiff by NDSEP (Image acquisition rate: 7.5Hz)	2.15 (std:0.03)	–	1.11 (std: 0.01)	4.56 (std: 0.02)	14.31 (std: 0.13)	22.13 (std:0.16) (45.19 Hz)
Neurofinder 03 (498 × 490) 613-neuron .tiff by NDSEP (Image acquisition rate: 7.5 Hz)	2.14 (std:0.11)	–	1.19 (std: 0.07)	2.76 (std: 0.68)	33.32 (std: 1.05)	49.42 (std:1.54) (20.24 Hz)
ALM (512 × 512) 69-neuron .tif by NDSEP (Image acquisition rate: 15 Hz)	3.95 (std:0.04)	11.53 (std: 0.09)	0.71 (std: 0.01)	1.35 (std: 0.01)	3.13 (std: 0.01)	20.67 (std:0.15) (48.38 Hz)

All of the execution time measurements were taken on a MacBook Pro laptop computer. The computer was equipped with a 2.5 GHz Intel Core i7 CPU, the Mac OS High Sierra 10.13.1 operating system, and 16 GB memory.

The experiments on execution time were performed on all of the four datasets employed in section 4.1 through section 4.3. Since image registration has already been applied in the two Neurofinder datasets, we disabled the Motion Correction actor in the experiments with these two datasets. For the simulated dataset, the two Neurofinder datasets, and the ALM dataset, average execution times were taken across 1,800, 2,245, and 11,189 frames, respectively. The results in Table 4 show average execution times *per frame* for each actor/dataset combination.

Generally, the Motion Correction actor dominated the overall execution time for the datasets in which the actor was used. The signal extraction actor exhibited the largest variation in execution time. We anticipate that this is because of the strong dependence of this actor's execution time on the number of detected neurons. Also, not only the number of detected neurons but also how active the neurons are affect the detection actor's execution time. As shown in Table 4, the total execution time, summed across all actors, was less than 22 ms per frame for all datasets that we experimented with, except for the Neurofinder 03 dataset, which has over 600 neurons detected. Considering the image acquisition rate, for all four datasets, NDSEP is shown to provide adequate performance for real-time operation without the need for expensive, cumbersome, or special-purpose computing hardware.

### 4.5. Comparison With Other Platforms

Using the simulated dataset and ALM dataset, we compared NDSEP-based motion correction with CaImAn-NoRMCorre (Non-Rigid Motion Correction) and also with ImageJ-SIFT (Scale Invariant Feature Transform). In addition, a neuron detection comparison was made among NDSEP-based neuron-detection, CaImAn-CNMF (CaImAn-Constrained Nonnegative Matrix Factorization), and CellSort (also known as PCA/ICA). For details on CaImAn-NoRmCorre, SIFT, CaImAn-CNMF, and CellSort, we refer the reader to Giovannucci et al. (2019), Lowe (2004), and Mukamel et al. (2009). Table 6 shows parameters used in the comparison approaches.

#### 4.5.1. Motion Correction Comparison

The Motion Correction comparison is made on the simulated dataset. In CaImAn-NoRMCorre, we experimented with both the rigid mode and non-rigid mode. Although CaImAn-NoRMCorre is not natively designed to correct rotations, rotations can be recognized when the grid size is small (the resolution is high). However, excessively small grid sizes make matches hard to find and greatly increase computational cost. In our experiments, we used an empirically determined grid size of [32, 32], which we found to provide an efficient balance between the aforementioned trade-offs. We also applied the cubic shifting method and a small overlapping region with a size of [16, 16]. All other parameters are set at the values recommended in Giovannucci et al. (2019). As with CaImAn-NoRMCorre, we tuned parameters in SIFT to maximize accuracy. Most of the parameter values that we used are those recommended in Lowe (2004). We set the maximal alignment error to two pixels to increase the accuracy, instead of 10% of the image size as recommended in Lowe (2004).

**Figure 9:**
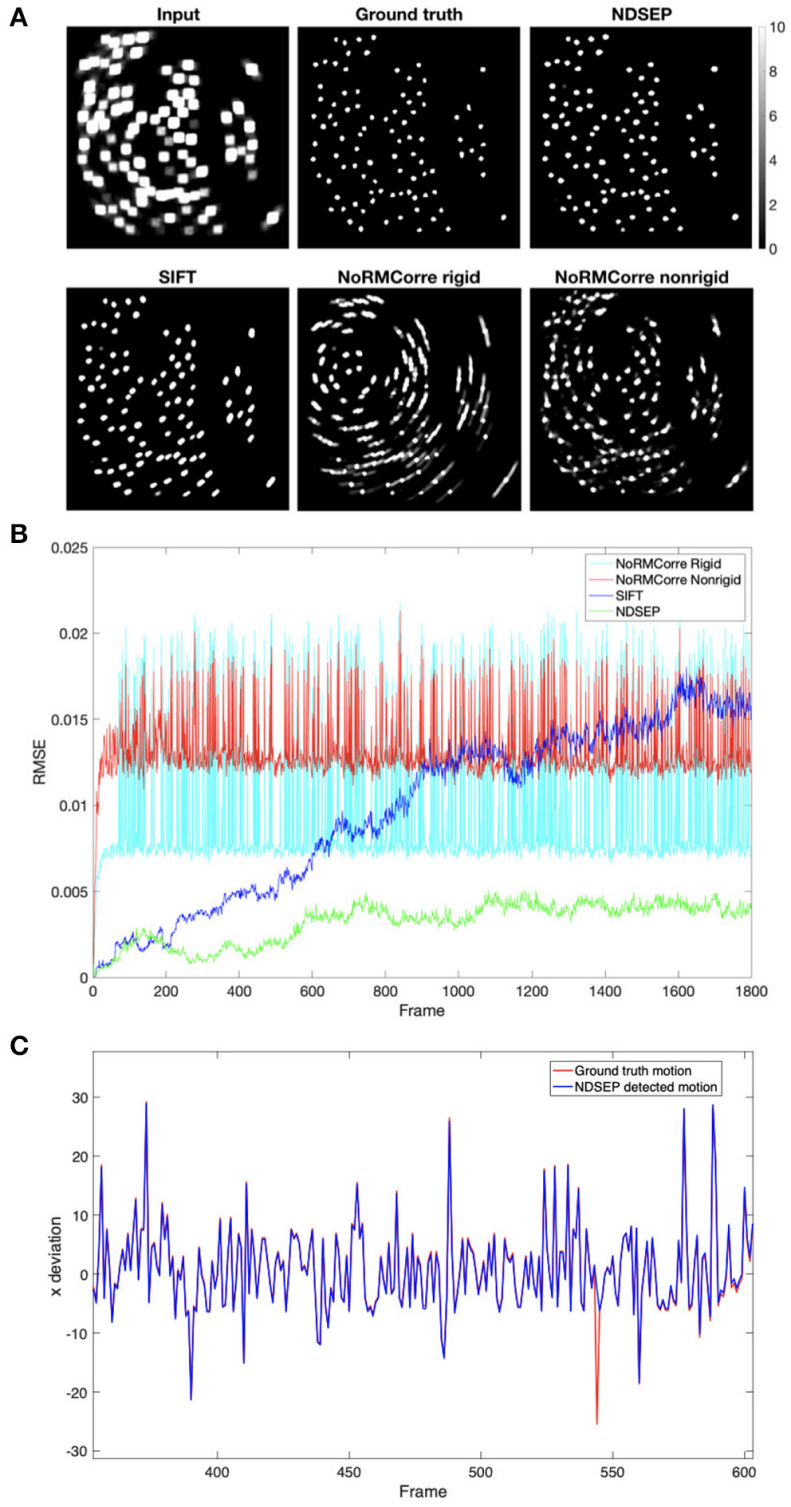
Motion correction comparison results on simulated dataset *P*_*rot*_ = 25 and α_*rot*_ = 6.3153 without noise. **(A)** Shows the uint8 [0, 255] meanmaps. The display range is [0, 10], where values greater than 10 are displayed in white. The input data is the meanmap of simulated data with motion but without noise. The ground truth is the meanmap of the no-motion simulated data. The remaining four images are the output meanmaps of NDSEP, SIFT, the rigid mode of CaImAn-NoRMCorre, and the nonrigid mode of CaImAn-NoRMCorre. **(B)** Shows the RMSE calculated by performing frame-by-frame comparison between the output of the four motion correction methods and the ground truth. **(C)** Shows the *x* movement, which is actual movement applied to simulated data, as a red line vs. movement detected by NDSEP motion correction as a blue line, including a program-detected failure in frame 544. Please note motions larger than the 10-pixel upper bound occur because of the rotations applied around the center of the image.

CaImAn-NoRMCorre is not able to align most of the rotations, as shown in Figure 9A, especially in the rigid mode. The high spike values in Figure 9B correspond to the non-corrected motions. The non-rigid mode has lower spikes, which correspond to rotations, than the rigid mode. However, the non-rigid mode exhibits higher error when correcting translation-only frames. SIFT achieves a relatively high accuracy for both translations and rotations, but Figure 9B shows that the root mean square error (RMSE) value increases as the number of frames increases. This means that the error accumulates and the accuracy drops as the system continues processing. This feature makes SIFT inefficient for our real-time motion correction context. Unlike CaImAn-NoRMCorre, NDSEP efficiently corrects nearly all simulated motion translations, shown in Figure 9C, and rotations while discarding the unusual uncorrected frames.

#### 4.5.2. Neuron Detection Comparison

In our comparison of neuron detection performance, experiments are performed on two datasets: simulated data and ALM data. On simulated data, two approaches, NDSEP-neuron detection and CaImAn-CNMF, are compared. On ALM data, three approaches, NDSEP-neuron detection, CaImAn-CNMF, and CellSort, are evaluated. To eliminate the influence of different motion correction approaches, the input datasets are first motion-corrected. In particular, the simulated data input is the motion-correction ground truth used to calculate the RMSE value in Figure 9B. The ALM input data is motion-corrected by CaImAn-NoRMCorre with the rigid mode following the parameters used to register the simulated data. When comparing different neuron detection approaches, we compare only the spatial component—that is, only the locations of the detected neurons.

In CaImAn-CNMF, the number of neurons *N*_*det*_ to be detected is predefined. In our experiments, for simulated data, we set *N*_*det*_ = 97 because there are a total of 97 neurons in the ground truth mask. *N*_*det*_ is set to 80, a little higher than the number of neurons in the ALM ground truth. This setting is used to increase the recall rate. The parameter τ of the Gaussian kernel is set to half the size of a single neuron. The resulting values for the simulated and ALM datasets are τ = 3.4 and τ = 8.0, respectively. The optional parameter *P* used for normalization by noise and user feed component centroids is disabled for simulated data, since the temporally autocorrelated noise we have cannot be removed in this way. Other parameters are tuned according to Giovannucci et al. (2019). In CellSort, which is only tested on the ALM dataset, the value of mu is set to 0.5, which enables the use of both temporal and spatial information for segmentation. Other CellSort parameters are set as recommended in Mukamel et al. (2009).

Table 5 shows that both CaImAn-CNMF and NDSEP detect all of the 97 neurons when the input frames are free from noise. However, for noisy input, the CaImAn-CNMF detection rate *CaImAn*−*CNMF*_*det*_ drops as the noise intensity increases, while NDSEP consistently provides 100% accuracy for both noise-free and noisy input. Thus, our experiments demonstrate that CaImAn-CNMF neuron detection is vulnerable to noise, while NDSEP is much more robust. Furthermore, as shown in Table 4, NDSEP requires significantly less processing time compared to CaImAn-NoRMCorre, SIFT, and CaImAn-CNMF.

**Table 5:** Neuron detection rate with different noise levels.

**Noise level**	**No noise**	**s01c05**	**s01c10**	**s01c15**	**s03c05**	**s03c10**	**s03c15**	**s05c05**	**s05c10**	**s05c15**
*CNMF*_*det*_(%)	100	97.9	89.7	74.2	92.8	89.7	74.2	85.6	74.2	68.0
*NDSEP*_*det*_(%)	100	100	100	100	100	100	100	100	100	100

**Table 6:** Parameters of other approaches.

**Parameter**	**Description**	**Value**
CaImAn-NoRMCorre on Simulated data rigid mode		
*bin*_*width*	Width of each bin	50
*max*_*shift*	Maximum rigid shift in each direction	[30, 30]
*init*_*batch*	Length of initial batch	10
CaImAn-NoRMCorre on Simulated data nonrigid mode		
*grid*_*size*	Size of non-overlapping regions	[16, 16]
*overlap*_*pre*	Size of overlapping region	[16, 16]
*shifts*_*method*	Method to apply shifts	cubic
*init*_*batch*	Length of initial batch	50
CellSort on ALM data		
mu	Parameter (between 0 and 1) specifying weight of temporal information in spatio-temporal ICA	0.5
*maxrounds*	Maximum number of rounds of iterations	1,000
CaImAn-CNMF on ALM data		
*K*	Number of components to be found	80
*tau*	Size of Gaussian kernel (half size of neuron)	8
*merge*_*thr*	Merging threshold	0.2
*min*_*SNR*	Minimum SNR threshold	1
CaImAn-CNMF on Simulated data		
*K*	Number of components to be found	97
*tau*	Size of Gaussian kernel (half size of neuron)	3.4

On the ALM dataset, CaImAn-CNMF correctly detects 53 among 69 neurons; the resulting recall is 76.81%, while the precision is 72.60%. CellSort segments 79 neurons, of which 57 are correct (true positives), and the recall and precision using CellSort are 82.61 and 72.15%, respectively. For NDSEP, the corresponding results (shown in section 4.3) are: recall = 72.46% and precision = 69.44%. From these results, we see that the accuracy of NDSEP neuron detection is comparable to other state-of-the-art approaches such as CaImAn-CNMF and CellSort.

## 5. Discussion

In this paper, we proposed a real-time neuron detection and neural activity extraction system called the Neuron Detection and Signal Extraction Platform (NDSEP). NDSEP uses a novel integration of dataflow-based design architecture and streamlined algorithms and software modules for real-time neural signal processing. The dataflow architecture of NDSEP provides sufficient flexibility to expand the system, experiment with design trade-offs, and manage complex constraints of real-time neuron detection and activity extraction (RNDAE) systems. Such constraints include those involving memory requirements and cost-effective deployment.

In an experiment based on simulated calcium imaging data, NDSEP effectively performed motion correction with mean errors of *x* and *y* displacement of <1 pixel and mean rotation error close to zero. NDSEP detected all active neurons and achieved a very high signal-to-noise ratio. For the Neurofinder database and for a real-world dataset, ALM, NDSEP achieved comparable results for detection, and the detected neurons demonstrated typical calcium transient patterns. In all of these experiments, the execution times were shorter than 25 ms, and NDSEP achieved real-time performance.

As presented in section 3, the key subsystems in NDSEP for neural signal processing are system parameter optimization (represented by the SetParams actor), motion correction, neuron detection, and neural signal extraction. We have developed and integrated initial versions of these subsystems through careful design, experimentation, and optimization to achieve real-time performance with reasonable system accuracy. However, many alternative combinations of algorithms, algorithmic parameter settings, and design optimization techniques can be applied to achieve the same general functionality as the current version of NDSEP, which involves the mapping of neural image streams into sets of neurons and their associated signals. These combinations represent a complex, largely unexplored design space, which involves trade-offs among real-time performance, neuron detection and signal extraction accuracy, and computational resource costs.

In addition to providing a complete system prototype for RNDAE, NDSEP provides a useful framework for investigating this design space and for developing further innovations in algorithms and systems for RNDAE. Such innovations could, for example, help to further increase the accuracy of neural signal extraction while maintaining real-time performance. Alternatively, they could help to reduce system costs without significantly sacrificing accuracy, thereby contributing to more cost-effective technologies for scientists, clinicians, or patient-users. The model-based design architecture of NDSEP, based on our application of dataflow design methods, helps to precisely formulate the aforementioned design space in terms of component subsystems (actors for RNDAE) and precise interfacing requirements between them. The modularity and abstract design of the NDSEP architecture greatly facilitate experimentation with alternative combinations of component algorithms, algorithm configurations, and hardware/software realizations of the algorithms.

Four general directions for future work emerge naturally from the properties described above of the NDSEP architecture and its utility in defining and exploring important design spaces for RNDAE system design. The first direction is exploration into new algorithms and implementations for the four key component subsystems. Examples of concrete topics in this direction include applying downsampling strategically in parts of NDSEP outside of motion correction, where it is already applied (see section 3.2.1). Another example is incorporating more sophisticated processes for parameter adaptation and optimization in the initialization mode of NDSEP, as motivated in section 3.3.2.

A second direction for future work is in applying the NDSEP platform to develop novel systems for precise neuromodulation. The current system will be part of a precise all optical closed-loop neuromodulation system that combines calcium image processing (the current system), prediction (predicting behavioral variables based on neural features), and neuromodulation (optogenetics). In our recent prior work, our team has developed pilot versions of prediction (Lee et al., 2017) and network-based feature extraction (Chen and Lin, 2018) for calcium imaging data. The primary design goal of NDSEP is real-time data processing. Existing optogenetics intervention permits millisecond-precision manipulation of genetically targeted neural populations (Häusser, 2014). In our future work, we will improve these pilot versions and integrate them with NDSEP. We expect that our future all-optical closed-loop neuromodulation system can achieve real-time performance above 10 Hz, providing neuroscientists an open-source, real-time neural decoding system that facilitates precise neuromodulation.

A third direction for future work is studying design optimization methods and trade-offs in NDSEP in the context of overall cost and performance in the enclosing neuromodulation systems.

A fourth direction for future work is support for higher image acquisition rates. Results are unpredictable if the speed of the system is slower than the acquisition rate. The designer must therefore optimize and test the system carefully to ensure that constraints imposed by the acquisition rate are satisfied. The dataflow-based system architecture facilitates these optimization and testing objectives. Our current system is designed for two-photon calcium imaging. The typical acquisition rate is 10–30Hz. Based on Table 4, the current implementation can handle such an acquisition rate. In the future, if we want to use NDSEP for high-speed calcium imaging with a 180–490 Hz sampling rate, hardware acceleration within the framework of dataflow-based design may be used.

On top of the four main directions described above, since NDSEP focuses on real-time computation using efficient detection algorithms, it may have difficulty detecting overlapping neurons. In addition to this, NDSEP can be extended to be enabled for one-photon calcium imaging with more noise. More comparisons to the state-of-the-art methods like OnACID should be made. Also, NDSEP does not include an actor for neuropil fluorescence contamination. We will address these limitations in our future work.

The NDSEP system developed in this study is an efficient, extensible system based on dataflow design for real-time neuron detection and neural activity extraction. We expect that the platform will enable real-time calcium imaging-based neural decoding, leading to precise neuromodulation.

## 6. Code Availability

The NDSEP software and documentation are available from [http://dspcad-www.iacs.umd.edu/bcnm/index.html].

## Data Availability Statement

Publicly available datasets were analyzed in this study. This data can be found here: https://github.com/codeneuro/neurofinder.

## Author Contributions

YL wrote the first draft of the manuscript and conducted experiments. JX wrote selected parts in subsequent drafts of the manuscripts and conducted experiments. EL and SS helped with integrating algorithms and conducting experiments. D-TL organized the data and provided datasets. RC and SB contributed to the conception and design of the study and revision. All authors contributed to the article and approved the submitted revision.

## Conflict of Interest

The authors declare that the research was conducted in the absence of any commercial or financial relationships that could be construed as a potential conflict of interest.
