# Supplementary material for: Real-Time Neuron Detection and Neural Signal Extraction Platform for Miniature Calcium Imaging
Source: Front Comput Neurosci. 2020 Jun 26;14:43. doi: 10.3389/fncom.2020.00043 (PMC7333463; doi:10.3389/fncom.2020.00043)
Supplement: Supplementary file 1 [file Data_Sheet_1.PDF]

Real-time Neuron Detection and Neural Signal Extraction Platform for Calcium Imaging

1 SUPPLEMENTARY DATA

1.1 Neurofinder data

Table S1. The information about the neurofinder data that we used neu (2016).

	Neurofinder 01	Neurofinder 03
Lab	Hausser Lab	Losonczy Lab
contributors	["Adam Packer", "Lloyd Russell"]	["Jeff Zaremba"]
animal	Mouse	Mouse
animal-state	Awake head-fixed	Awake head-fixed
experiment	Drifting grating visual stimuli	Hidden reward spatial navigation
method	Two-photon raster	Two-photon raster
indicator	GCaMP6s	GCaMP6f
region	V1	dHPC CA1
pixels-per-micron	0.8	1.7007
rate-hz	7.5	7.5
dimensions	[512, 512, 2250]	[498, 490, 2250]

2 SUPPLEMENTARY TABLES AND FIGURES

REFERENCES

[Dataset] (2016). The neurofinder chanllenge

Table S2. Summary of variables and symbols.

Variable	Section	Description and Value
$\{n_1, n_2, \ldots, n_m\}$	1	Neuron masks set
G	2.2	Dataflow graph
X	2.2	Set of actors
E	2.2	Set of edges
e	2.2	An edge in E
p	3.1	Output port of the subinit graph
$n(p)$	3.1	The number of ordered pairs associated with p
$A_i(p)$	3.1	An actor in the body graph
$P_i(p)$	3.1	A parameter of actor $A_i(p)$
F_c	3.2.1	The current frame
F_s	3.2.1	The shifted frame
F_r	3.2.1	The reference frame
C_1, C_2	3.2.1	Computed correlation in Translation Only (C_1)/Euclidean (C_2) mode
$\tau = \{\tau_1, \tau_1\}$	3.2.1	Threshold in Translation Only (τ_1)/Euclidean (τ_2) mode
$p(\tau)$	3.2.1	Empirically defined parameter for τ_1 and τ_2, $p(\tau_1) = 2$, $p(\tau_2) = 10$
I	3.2.2	The current pixel's intensity
n_d	3.2.3	The number of detected neurons
δ	3.2.3	Neuron detection matrix
`thresholdStep`	3.2.3	The minimum intensity difference between the inside and outside of a blob
A_{min}, A_{max}	3.2.3	The minimum/maximum sizes of the blobs to detect
η	3.2.3	A set of neurons identified by neuron detection
F_{mc}	3.2.4	A motion-corrected image frame
β	3.2.4	Firing output vector
L	3.2.4, 3.3.3	Total number of image frames in the input video sequence
T_n	3.3.2	Pre-determined target number of neurons, $T_n = 5$
V	4.1	Membrane potential
V_{rest}	4.1	Rest potential
λ	4.1	Membrane time constant
P_{rot}	4.1	Rotation occurrence probability
α_{rot}	4.1	Rotation range
ϕ	4.1	Detected neuron
r	4.1	Some region in the frame
F	4.1	Average pixel intensity in I
R	4.1	Average pixel intensity in r
R_s	4.1	Average correlation coefficient across all neurons
$\rho(\phi)$	4.1	Correlation between $\Delta F/F$ of r and the ground-truth spike train of ϕ
R_n	4.1	The average value of $\rho(\phi)$
M_x, M_y	4.1	x-displacement, y-displacement
M_{rot}	4.1	angle error
$Rate_{fail}$	4.1	Failure rate of motion correction
$mean(\alpha_{rot})$	4.1	mean rotation error
